# Novel nano-vehicle for delivery and efficiency of anticancer auraptene against colon cancer cells

**DOI:** 10.1038/s41598-020-58527-0

**Published:** 2020-01-31

**Authors:** Nazila Jalilzadeh, Naser Samadi, Roya Salehi, Gholamreza Dehghan, Mehrdad Iranshahi, Mohammad Reza Dadpour, Hamed Hamishehkar

**Affiliations:** 1 Faculty of Natural Sciences, University of Tabriz, Tabriz, Iran; 2 Department of Biochemistry and Clinical Laboratories, Faculty of Medicine, Tabriz University of Medical Sciences, Tabriz, Iran; 3 Drug Applied Research Center, Tabriz University of Medical Sciences, Tabriz, Iran; 4 Department of Medical Nanotechnology, Faculty of Advanced Medical Sciences, Tabriz University of Medical Sciences, Tabriz, Iran; 5 Faculty of Pharmacy, Mashhad University of Medical Sciences, Mashhad, Iran; 6 Department of Horticulture, Faculty of Agriculture, University of Tabriz, Tabriz, Iran

**Keywords:** Drug delivery, Polymer synthesis

## Abstract

The aim of this study is to devise, prepare and characterize nano encapsulated auraptene (AUR) and evaluate cytotoxic and apoptotic effects on HT-29 colon cancer cells. Herein, AUR nano formulations were prepared by triblock (PCL-PEG-PCL) and pentablock (PLA-PCL-PEG-PCL-PLA) biodegradable copolymers in order to increase AUR bioavailability as an anticancer agent. The preparation of nano particles (NPs) was done with rotor stator homogenization (RSH) and Ultrasonic homogenization (USH) methods. The physicochemical characteristics of prepared nanoparticles (NPs) were studied using HNMR, FTIR, GPC, DLS and SEM techniques. The smaller hydrodynamic size (110 nm) and polydispersity index (PDI: 0.288) as well as higher cellular uptake (89%) were observed in PB NPs rather than TB NPs. The highest cytotoxic and apoptotic effects were observed in AUR loaded PB NPs compared to AUR loaded TB NPs and free AUR obtained by MTT assay, cell cycle arrest, Annexin V-FITC, DAPI staining and RT-PCR techniques. Real time PCR results indicated that Bax /Bcl2 expression ratio as an apoptosis predicting criterion, in free AUR, AUR loaded TB and AUR loaded PB have increased 6, 9 and 13 times, respectively (p value < 0.05). In conclusion, using biodegradable nano-vehicles for sustained delivery of natural anti-cancer compounds may open new perspectives for treatment of cancer patients.

## Introduction

Although chemotherapy is the most common strategy for cancer treatments, using individual chemotherapy in eradication and elimination of tumors is in lined with a range of side effects. In spite of many efforts, the side effects of anticancer drugs are still a major issue in chemotherapy for malignant diseases^1^. Nano medicine and nano drug delivery may help opening of novel ways toward efficient safe targeted therapies. The goal of nanoparticle based drug delivery systems is to achieve a more effective therapeutic index with the least side effects^2^. Based on cancer statistics reports colon cancer is one of the major causes of cancer deaths in the world^3^ and induction of apoptosis is a mechanism of tumor suppression that eliminates potentially cancerous and the cells with DNA damage, which is a mechanism that cancer cells escape^2,4^. A group of proteins, regulate apoptosis^5^ Bcl2 family is one of the responsible group of proteins which are key regulators of apoptosis through the internal pathway (mitochondrial)^5,6^. Bax with pro-apoptotic and Bcl2 with anti-apoptotic properties, genes are capable of predicting the progression of apoptosis. Coumarins are compounds that can be found abundantly in the nature with many therapeutic effects. Large amount of studies has reported apoptosis inducing effects of coumarins. Prenyl oxycumarins are a category of coumarins belonging to the family of Rutaceae and Apiaceae^7^. The most abundant prenyl oxycumarin in the nature is granyl oxycoumarin or Auraptene (AUR), which is well known today for its many pharmaceutical properties including high anti-cancer, antimicrobial, anti-fungal, anti-inflammatory, antioxidant, antibacterial for the tooth^8^ liver and nerve protective, and anti-hypertension properties^9,10^. Several studies have reported that Auraptene has the potential of apoptosis induction in breast and gastric cancer cells. The main limitations of these compounds, including AUR, is their low solubility in aqueous solutions that leads to low bio distribution and delivery to targeted sites^11^. Nano-size drug particles can solve the problem and penetrate into solid tumors, due to the same nano-size property^12,13^. Therefore, along with the necessity for effective cancer therapy, high cellular uptake of NPs containing drugs may enhance the internalization of chemicals into cancer cells which leads to the improvement of anticancer efficacy^14^. In the recent years, amphiphilic copolymers with hydrophobic and hydrophilic segments have attracted considerable attention due to their unique phase behavior in the aquatic environment and potential applications for drug delivery systems^15–18^. Polycaprolactone (PCL), poly lactic acid (PLA) and polyethylene glycol (PEG) are biodegradable polymers certified by the world food and drug administration (FDA)^19,20^. Polycaprolactone is a valuable biodegradable polymer that some enzymes including pseudomonas lipase (PS) can greatly increase this polymer degradation. Furthermore because of lipophilic nature, this polymer is able to entrap hydrophobic drugs through hydrophobic interactions^21^. PCL-PEG-PCL(triblock, TB) and PLA-PCL-PEG-PCL-PLA (pentablock, PB) copolymers have significant properties, such as long-term targeted drug delivery and ability to carry both hydrophobic and hydrophilic drugs^22^. These polymers can form micellar structures with an internal hydrophobic surface and an external hydrophilic surface in the aqueous solutions and the heart of the micellar structure can act as a reservoir for compounds with very low solubility in water. Micellar structures have some characteristics, such as small size in nanometer range and good thermodynamic stability in physiological conditions^18^.

In this study we aimed to increase therapeutic efficacy of AUR by providing an advanced vehicle for delivery of the agent with markedly higher bioavailability and bio distribution. Therefore, we first designed nano particles (self-assembling micelles) with using biodegradable PCL-PEG-PCL and PLA-PCL-PEG-PCL-PLA copolymers. Then nano particles size, zeta potential and chemical structure were studied. Furthermore, apoptotic effects of AUR loaded nano particles on HT-29 colon carcinoma cells were investigated.

## Results

### Polymer synthesis and characterization

**Figure 1:**
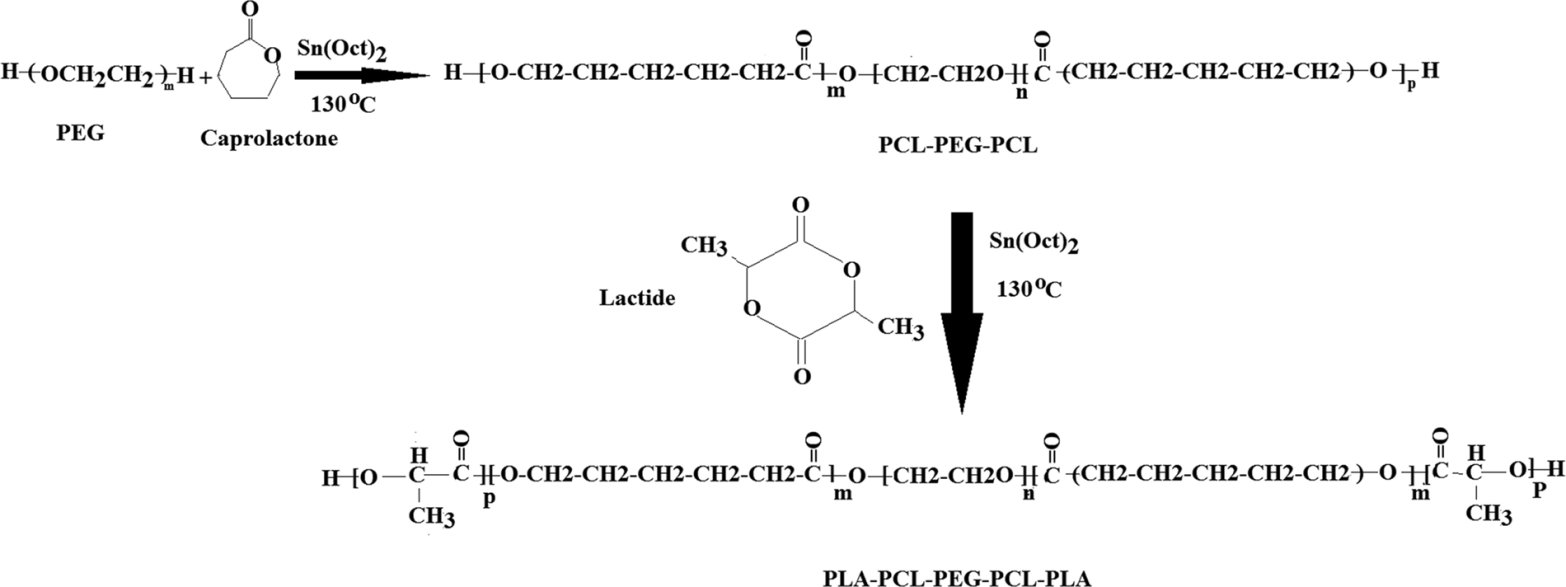
Schematic synthesis root of PCL-PEG-PCL triblock (TB) and PLA- PCL-PEG-PCL-PLA (PB) copolymers.

Triblock and pentablock copolymers were produced by sequential ring opening polymerization of ε-caprolactone and lactide. In the first step, triblock copolymer of PCL-PEG1000-PCL with PCL to PEG ratio of 2/1 was synthesized by ring opening polymerization of ε-caprolactone in the presence of PEG and predetermined amount of stannous octoate^23^. In the next step, PCL-PEG-PCL triblock copolymer served as macroinitiator for the synthesis of pentablock (PLA-PCL-PEG-PCL-PLA) copolymer by ring opening polymerization of D, L lactide (Fig. 1). Synthesized copolymers were characterized with HNMR and FTIR techniques.

#### HNMR studies of TB and PB copolymers

**Figure 2:**
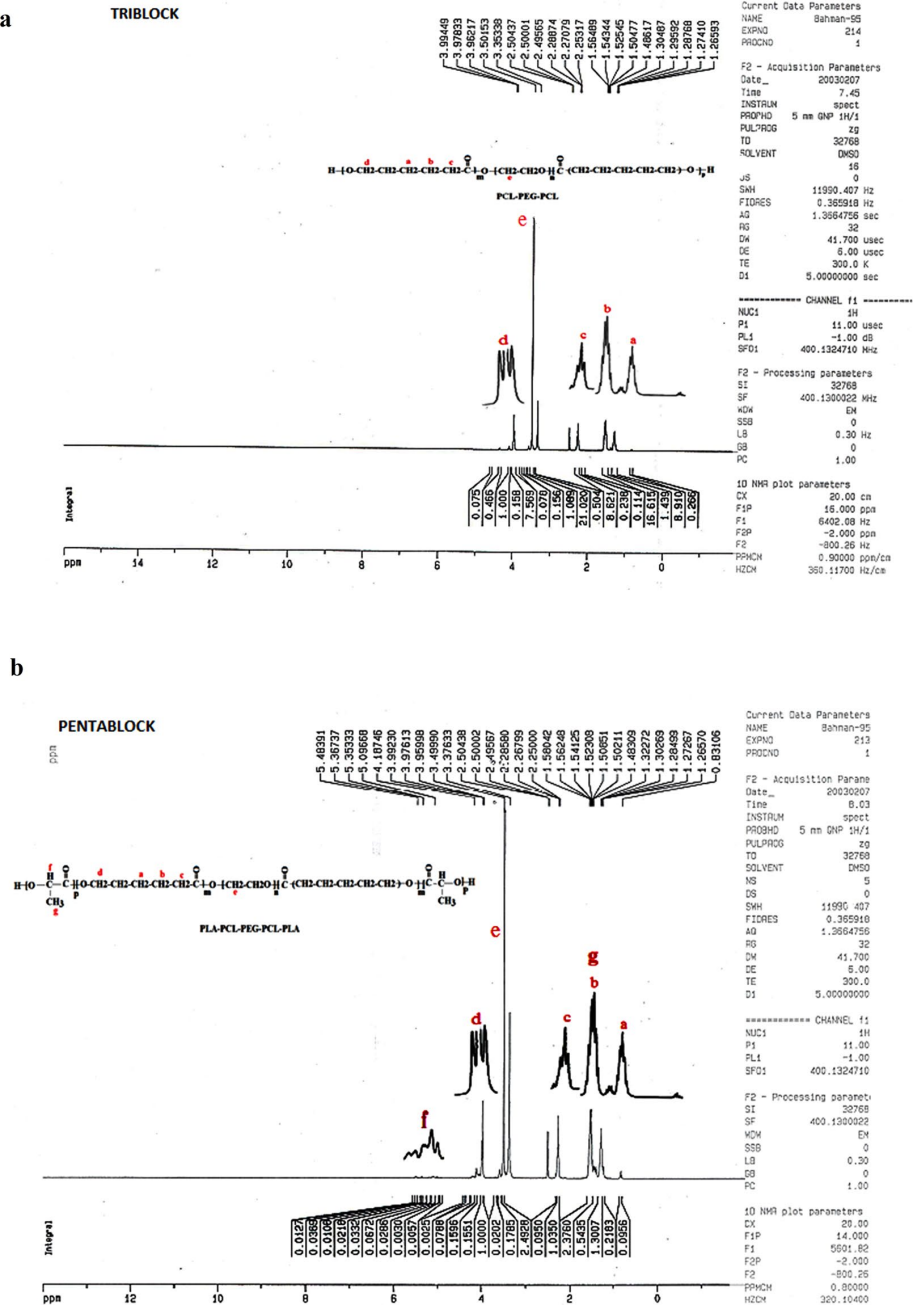
HNMR spectroscopy of (**a**) PCL-PEG-PCL triblock (TB) and (**b**) PLA- PCL-PEG-PCL-PLA (PB) copolymers.

Figure 2 shows HNMR spectra of synthesized TB and PB copolymers. In the interpretation of graphs obtained from HNMR of TB and PB copolymers, protons which associated with the PLA block appeared in the region of 1.5 (g in Fig. 2b, CH–CH_3_) and 5.4 (f in Fig. 2b, CH–CH_3_) ppm. Protons appeared in the 3.4 (e in Fig. 2a,b, CH_2_–CH_2_–O) ppm region are related to all protons of PEG. Caprolactone protons appeared in regions of 1.3 (a in Fig. 2a,b, O–CH_2_– CH_2_– CH_2_– CH_2_–CH_2_–C=O), 2.3 (b in Fig. 2a,b, O–CH_2_– CH_2_– CH_2_– CH_2_–CH_2_–C=O), 2.5 (c in Fig. 2a,b, O–CH_2_– CH_2_– CH_2_– CH_2_–CH_2_–C=O) and 4 (d in Fig. 2a,b, O–CH_2_– CH_2_– CH_2_– CH_2_–CH_2_–C=O) ppm^23,24^.

#### Molecular weight determination of TB and PB copolymers

The GPC chromatogram of PCL-PEG-PCL and PLA-PCL-PEG-PCL-PLA copolymers were shown in Figs. S1 and S2. Polydispersity indexe (PDI) values of PCL-PEG-PCL and PLA-PCL-PEG-PCL-PLA copolymers were 1.278 and 1.267, respectively. Also, average number (Mn) and average molecular weight (Mw) were obtained for synthesized PCL-PEG-PCL copolymer were 4243 and 5425 while for PLA-PCL-PEG-PCL-PLA copolymer were 4634 and 5871 g/mol, respectively.

#### FTIR studies

**Figure 3:**
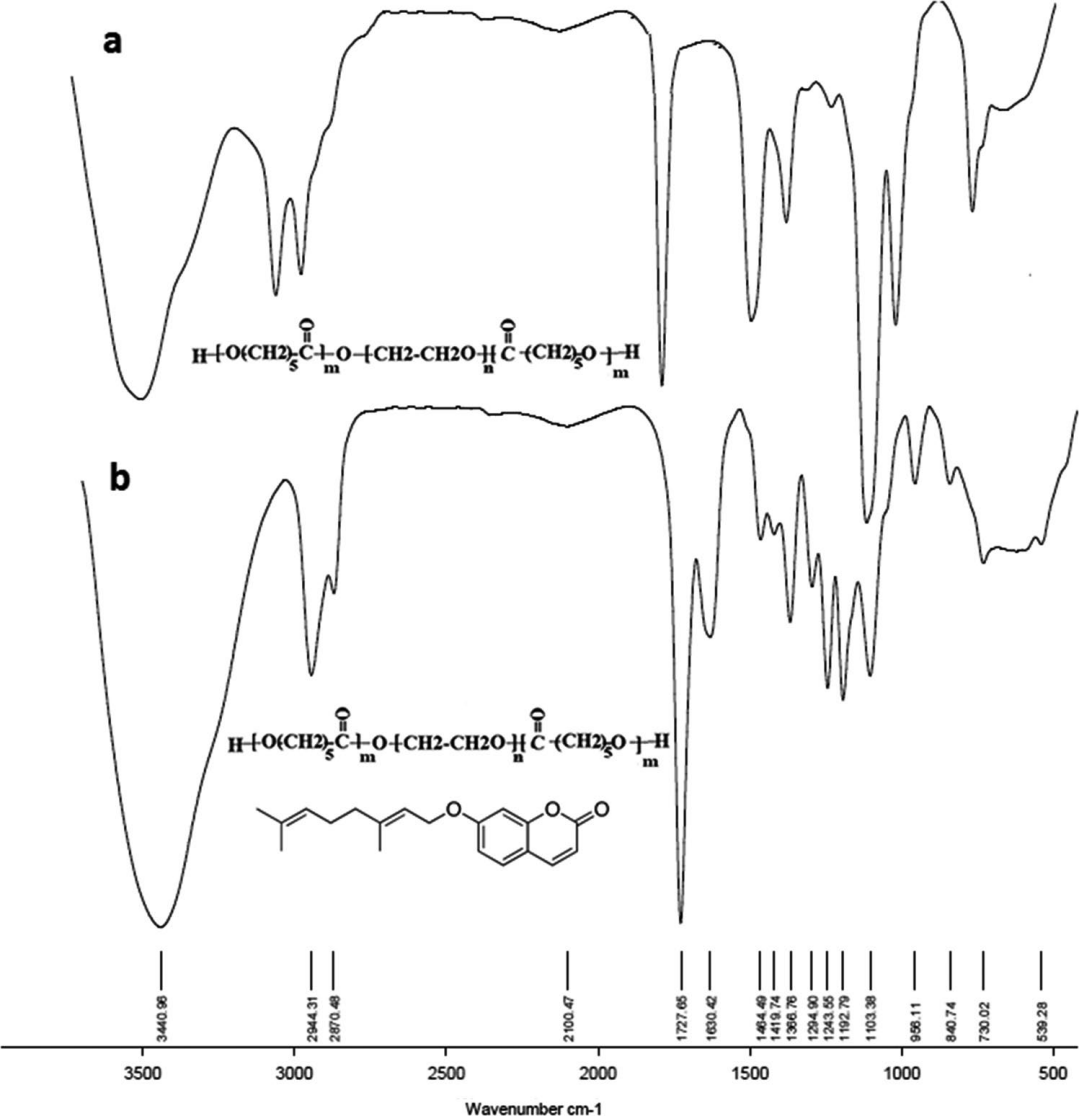
FTIR spectroscopy of (**a**) PCL-PEG-PCL triblock (TB) and (**b**) auraptene-loaded PCL-PEG-PCL triblock (AUR-TB) nanoparticles.

**Figure 4:**
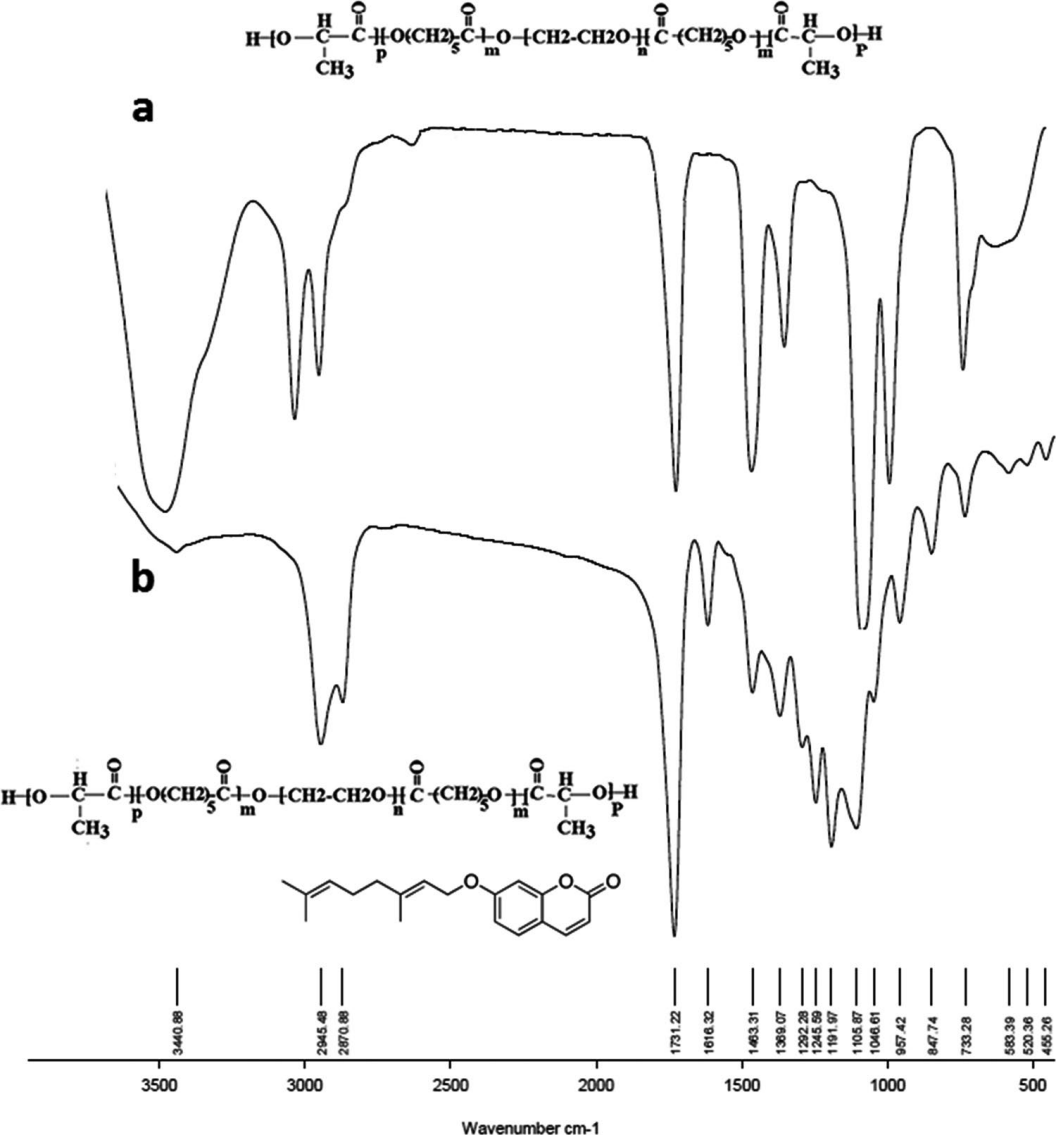
FTIR spectroscopy of (**a**) PLA-PCL-PEG-PCL-PLA pentablock (PB) and (**b**) auraptene-loaded PLA-PCL-PEG-PCL-PLA pentablock (PB) nanoparticles.

In FTIR spectra, chemical structure is identified based on the functional groups of the compound. In the case of PB and TB copolymers, since the TB polymer is a subset of PB, all observed peaks in TB are also fully observed in PB spectrum (Figs. 3 and 4). Figure 3 shows FT-IR spectra of TB copolymer (Fig. 3a) and AUR loaded TB (Fig. 3b). Stretching vibrations of the ester carbonyl group (C=O) appeared at 1736 cm^−1^. The bands at 1250–1100 cm^−1^ are associated to the C–O–C stretching vibrations of the repetitive –OCH_2_CH_2_ units of PEG. The absorption band at 3442 cm^−1^ is related to terminal –OH groups. All the C–H stretching bonds are centered at 2980–2800 cm^−1^ ^24,25^. After AUR loading, the presence of new peaks at 1630 (in AUR loaded TB spectrum) (Fig. 3b) and 1618 cm^−1^ (in AUR loaded PB spectrum) (Fig. 4b) were related to vinyl bonds of AUR that proves the encapsulation of drug in both copolymers.

### NP preparation and characterization

#### Nano particles size, zeta potential and morphology

**Figure 5:**
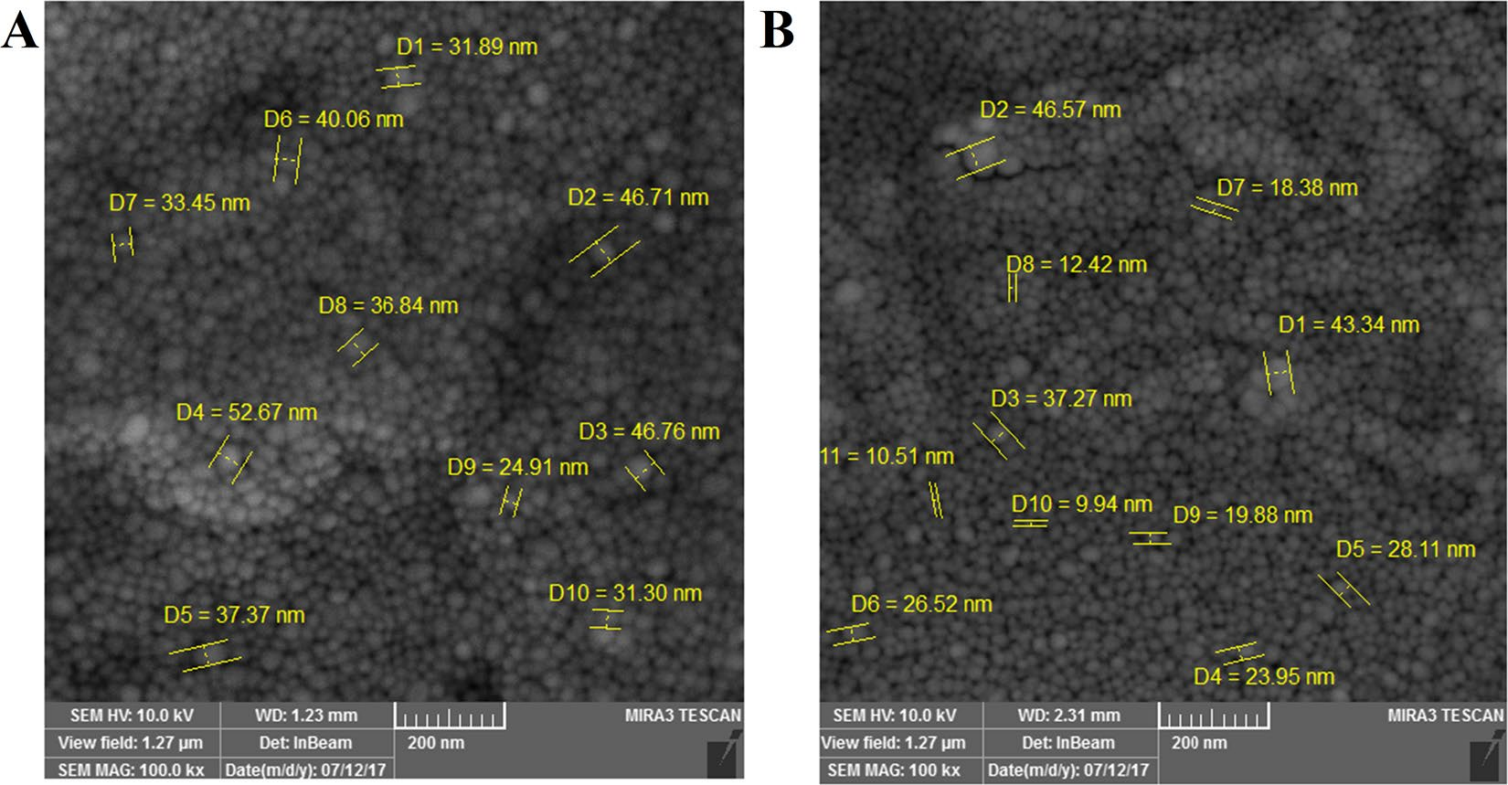
Size and morphology results of PCL-PEG-PCL triblock (TB) nanoparticles prepared by (**A**) rotor stator homogenization (RSH) and (**B**) Ultrasonic homogenization (US) methods determined by Scanning Electron Microscopy (SEM).

**Figure 6:**
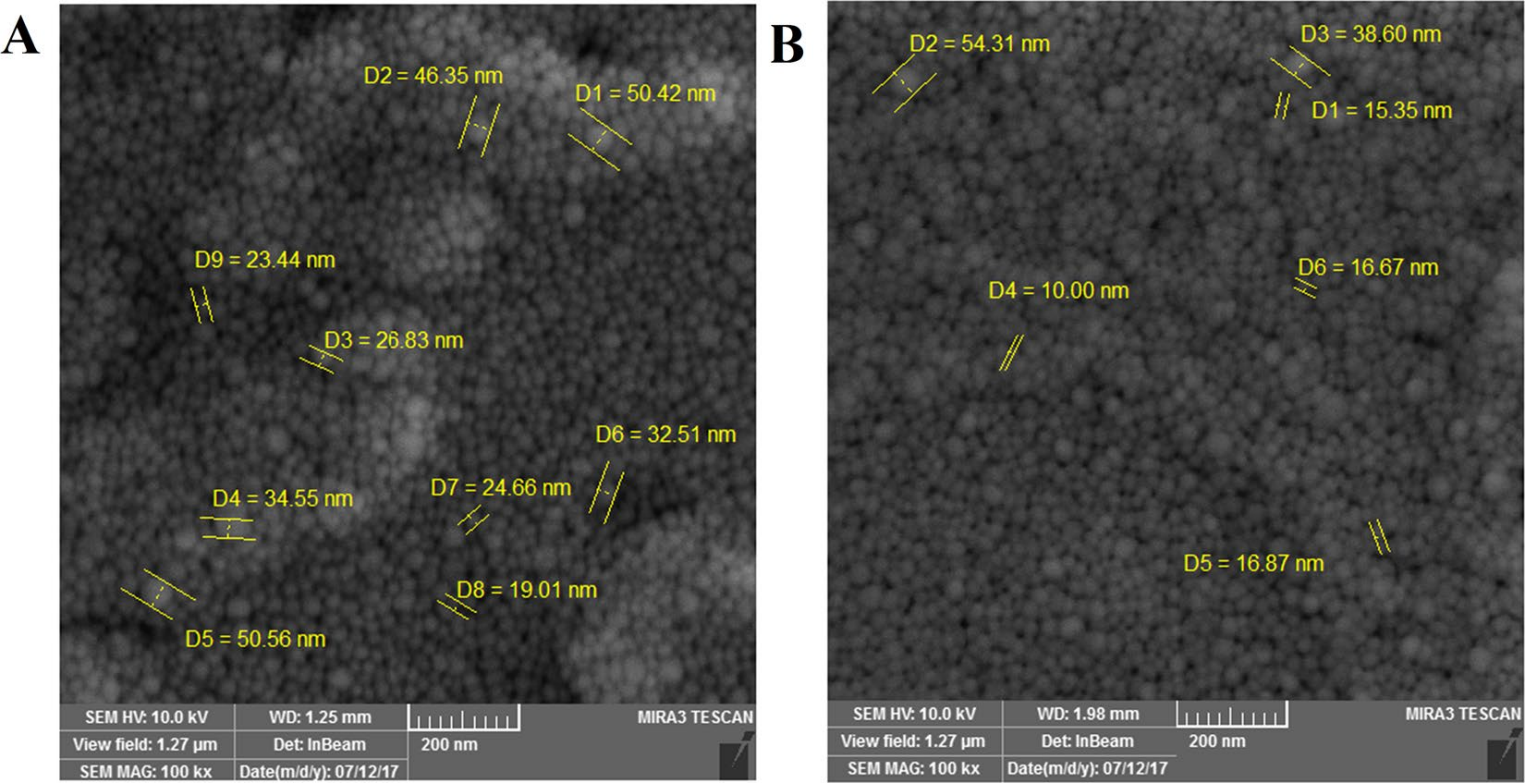
Size and morphology results of PLA- PCL-PEG-PCL-PLA (PB) nanoparticles prepared by (**A**) rotor stator homogenization (RSH) and (**B**) Ultrasonic homogenization (US) methods determined by Scanning Electron Microscopy (SEM).

Table 1 summarized the size distribution, polydispersity index and zeta potential of TB and PB NPs prepared by RSH and USH methods, measured by DLS technique. Blank PB and TB NPs size prepared by RSH method were 170 and 308 nm, while size of NPs prepared by USH method were 109.8 and 314.5 nm, respectively. PDI values of PB and TB NPs prepared by RSH method were 0.805 and 0.675 while, this value for NPs prepared by USH methods were 0.288 and 0.192, respectively (Figs. S3–S10). Surface charge of TB and PB NPs which were prepared by RSH method were −26.4 mV and −19.6 mV and for TB and PB NPs which were prepared by USH method were −17.5 mV and −9.26 mV. Due to the superiority of USH compare to RSH method in terms of smaller size, narrower PDI and lower negative surface charge, we used USH method in rest of this study. Figures 5 and 6 show the SEM images of TB and PB NPs prepared by both RSH and USH methods. As it has been seen in the SEM images revealed that NPs real size were in the range of 10 nm to 50 nm also NPs had homo-dispersed spherical morphology.

**Table 1:** Triblock (TB) and pentablock (PB) Nano-particles properties, prepared by two high energy methods.

USH method				RSH method		
Particle name	PDI	Zeta potential	Hydrodynamic size	PDI	Zeta potential	Hydrodynamic size
TB	0.192	−9.26	314.5 nm	0.675	−19.6	308 nm
PB	0.288	−17.5	109.8 nm	0.805	−26.4	170 nm

### Auraptene loading capacity and entrapment efficiency

Auraptene-loaded TB and PB NPs with different auraptene to polymers ratio were prepared by USH method. Entrapment efficiency (EE) and Loading capacity (LC) parameters of both AUR loaded TB and PB NPs were evaluated in three different polymers to drug ratio (formulation) (Table 2). Results showed that LC and EE values for formulation type I (polymer to drug ratio of 2 to 1) were 71% and 35.5% for TB and 91% and 45.5% for PB copolymers, respectively. These values for formulation type II (polymer to drug ratio of 5 to 1) were 89% and 17.8% for TB and 92% and 18.4% for PB copolymers, respectively. In formulation type III (polymer to drug ratio of 10 to 1) these parameters increased to 94% and 9.4% for TB and 95% and 9.5% for PB copolymers. Based on these findings due to the higher LC and acceptable EE of both TB and PB copolymers obtained for formulation type I, it was chosen for rest of the study. The average size and polydispersity index (PDI) of AUR loaded TB NPs was 265 nm and 0.758 while these values at the same condition, for AUR loaded PB NPs were 153 nm and 0.533, respectively (Figs. S11 and 12). After AUR encapsulation, the zeta potential of TB NPs increased from −9.26 to −16.4 mV and the zeta potential of PB NPs slightly decreased from −17.5 to −15 mV (Figs. S13 and 14) (Table 3).

**Table 2:** Loading capacity and encapsulation efficiency results of auraptene nano formulations.

Formulation type	Type I		Type II		Type III	
Parameter	LC	EE	LC	EE	LC	EE
Triblock	35.5%	71%	17.8%	89%	9.4%	94%
Pentablock	45.5%	91%	18.4%	92%	9.5%	95%

Formulation type I: polymer ratio to AUR: 2 to 1.

Formulation type II: polymer ratio to AUR: 5 to 1. Formulation type III: polymer ratio to AUR: 10 to 1.

**Table 3:** AUR loaded TB and PB NPs properties, prepared by ultrasonic homogenization (USH) method.

AUR loaded NPs			
Particle name	PDI	Zeta potential (mV)	Hydrodynamic size (nm)
TB(AUR)	0.758	−16.4	265
PB(AUR)	0.53	−15	153

### Auraptene release

**Figure 7:**
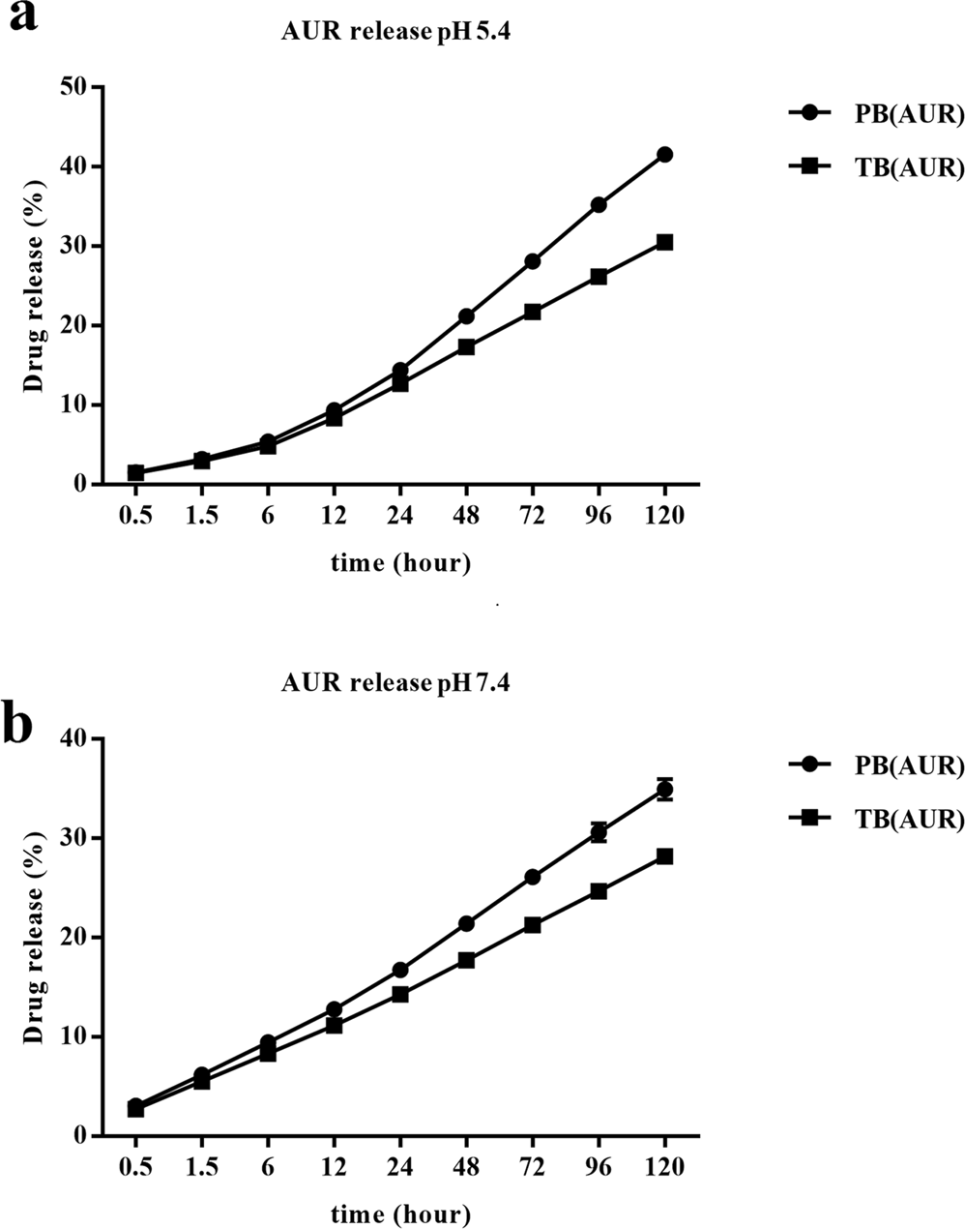
Cumulative release of auraptene from auraptene-loaded PCL-PEG-PCL triblock (AUR-TB) nanoparticles and auraptene-loaded PLA-PCL-PEG-PCL-PLA pentablock (PB) nanoparticles at pH values of (**a**) 5.4 and (**b**) 7.4 at 37 °C for about 120 hours.

In the current study, the release of AUR from TB and PB NPS was studied over 120 h and the controlled-release potential was assessed (Fig. 7). The release data generated in this work indicated that in pH 7.4 environment over 24 h only 16.74% and 14.29% of the drug were released from AUR loaded PB and AUR loaded TB NPs respectively. Which in pH 5.4 environment AUR release from AUR loaded PB and AUR loaded TB NPs at the same time were 14.39% and 12.76% respectively. In pH 5.4 environment after 120 h, 40.61% and 30.54% amount of AUR released from AUR loaded PB and AUR loaded TB NPs, respectively. At the same period, in pH 7.4 environment, 28 and 35% of AUR was released from TB and PB NPs, respectively. These data revealed that the prepared nano formulations exhibited sustained release profile with a slight pH-responsive pattern. The mean level of drug release was higher in the AUR loaded PB than it was in the AUR loaded TB NPs (P < 0.05).

### Intra cellular uptake of nanoparticles

**Figure 8:**
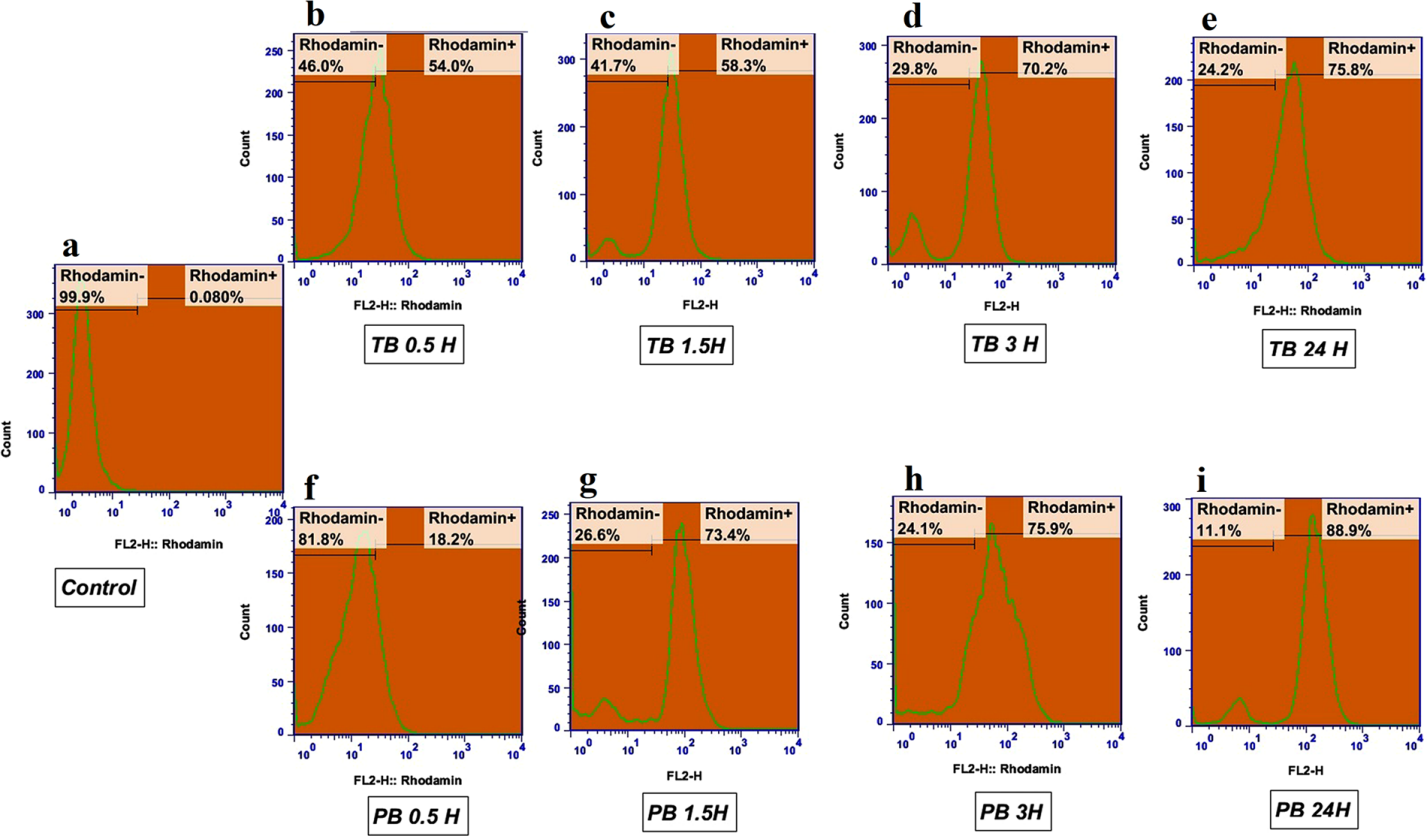
Rhodamine b labelled PCL-PEG-PCL Triblock (TB) and PLA- PCL-PEG-PCL-PLA pentablock (PB) nanoparticles uptake by HT-29 cells after different exposure times of 0.5, 1, 2, 3 and 24 h assessed by flow-cytometry.

**Figure 9:**
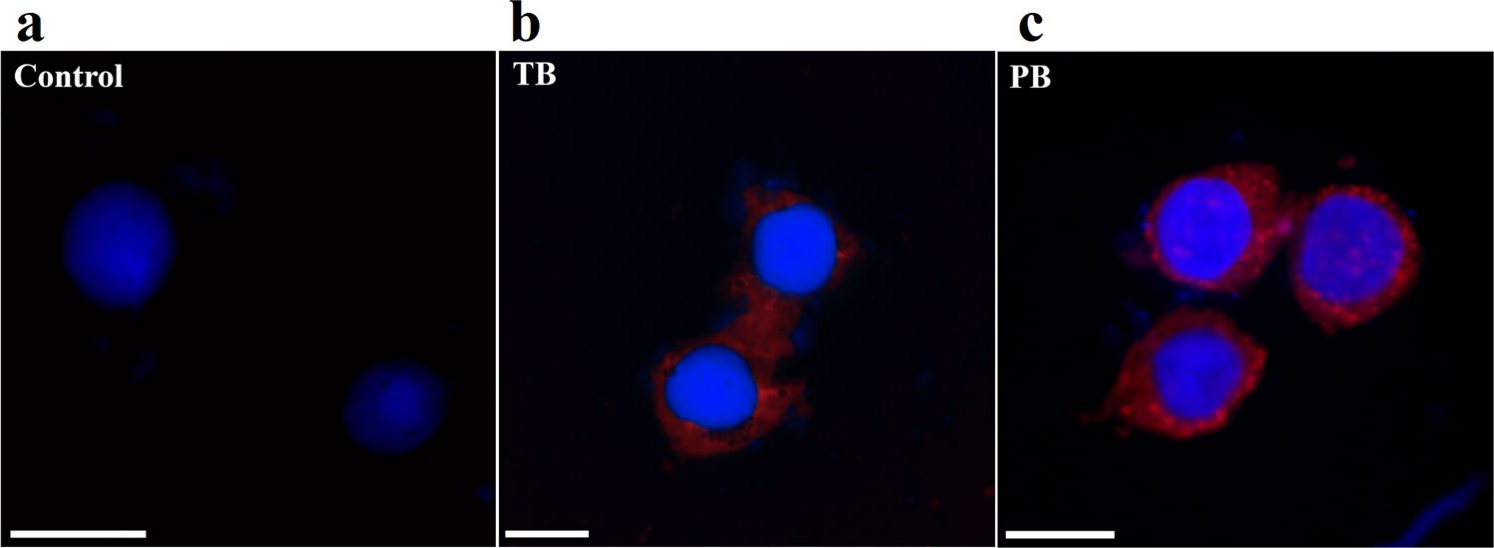
Cellular uptake of DAPI and rodamine b stained (**a**) untreated control, (**b**) PCL-PEG-PCL Triblock (TB) and (**c**) PLA- PCL-PEG-PCL-PLA pentablock (PB) nanoparticles in MCF-7 cells which were performed by Epi fluorescence microscope.

Cellular uptake of rhodamine b loaded TB and PB NPs were studied in predetermined time intervals (0.5 h to 24 h) (Fig. 8). Untreated cells were considered as control group, and the rest of the groups were compared against control. Cellular uptake of rhodamine b loaded TB and PB NPs after 0.5 h of incubation were 54 and 18% which these values increased to 58 and 73% after 1.5 h of incubation. Based on the results obtained from cellular uptake studies, both rhodamine b loaded TB and PB NPs showed high cellular absorption in early hours. TB and PB NPs uptake reached to 76 and 89%, respectively over 24 h incubation times. Cellular uptake studies which were performed by Epi fluorescence microscope confirmed results of flow cytometry studies (Fig. 9).

### Cytotoxicity of AUR, AUR loaded PB and AUR loaded TB

**Figure 10:**
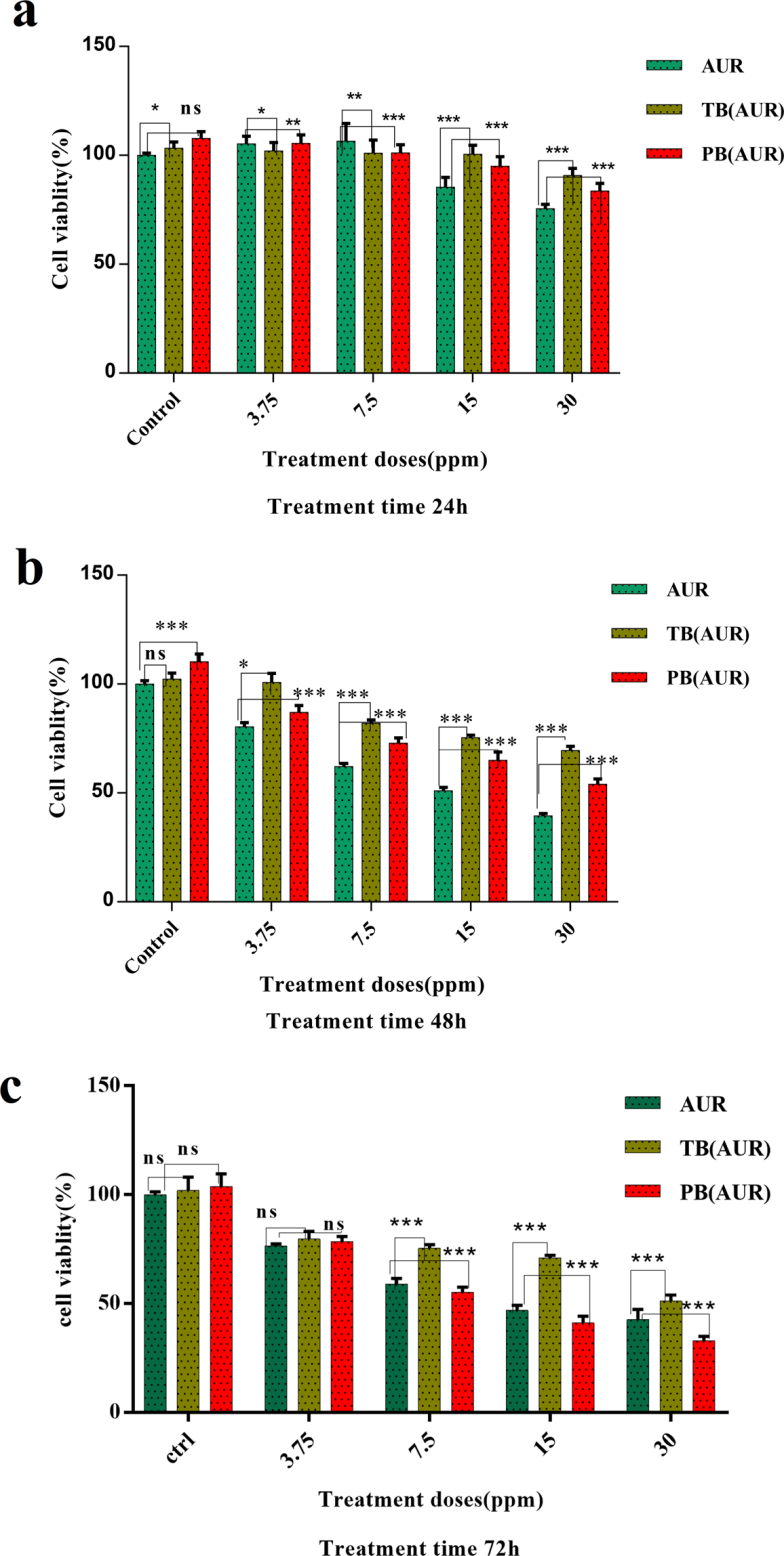
HT-29 cell growth inhibition rates from left to right in graph after exposure for (**a**) 24, (**b**) 48 and (**c**) 72 hours of treatment to different concentrations of free auraptene (AUR) (1st column), auraptene-loaded PLA-PCL-PEG-PCL-PLA nanoparticles (PB-AUR) (2nd column), and auraptene-loaded PCL-PEG-PCL nanoparticles (TB-AUR) (3rd column). The results were expressed as mean ± SD from three independent experiments. *p < 0.05, **p < 0.01 and ***p < 0.01.

Cytotoxicity of AUR, AUR loaded TB and AUR loaded PB in HT-29 colon cancer cells were investigated using MTT assay. Results (Fig. 10) revealed that IC_50_ value of AUR, after 48 and 72 h of exposure to HT-29 cells were 15 µg.mL^−1^, and 13.03 µg.mL^−1^ respectively. In AUR loaded TB, over 48 h of incubation, the IC_50_ value wasn’t observed at dose range was selected for this study (3.75–30 µg.mL^−1^) and over 72 h incubation the IC_50_ was 31.9 µg.mL^−1^. The IC_50_ value of AUR loaded PB after 48 and 72 h of incubation to HT-29 cells were 30 µg.mL^−1^ (100 μm), and 10.01 µg.mL^−1^ (33 μm), respectively. Cell viability results obtained by MTT assay after 24, 48 and 72 h of treatment among all treatment groups are statistically significant (p value < 0.05) except 3.75 ppm dose treatment group for 72 h.

Also HT-29 cells were exposed to high doses (1000 µg.mL^−1^) of blank TB and PB NPs, it was shown that both TB and PB NPs were completely safe and any cytotoxic effect wasn’t observed.

### Apoptosis assessed by flow cytometry and DAPI staining

**Figure 11:**
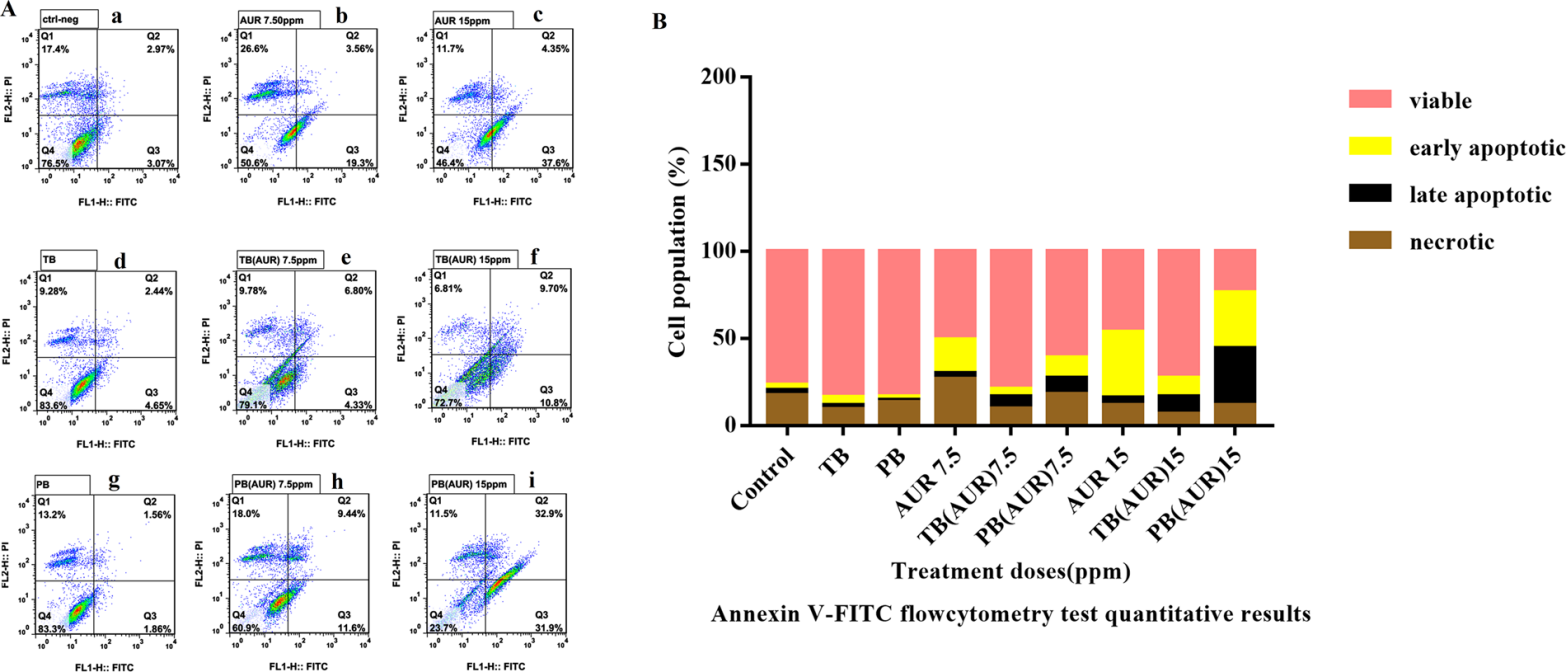
Annexin V and PI staining was used to identify viable cells (annexin V−, PI−), early apoptotic cells (annexin V+, PI−), late apoptotic (annexin V+, PI+) and necrotic cells (annexin V−, PI+). (**A**) The apoptotic effects of cells, determined by flow cytometry after 12 h in HT-29 cells for (a) untreated cells as a control, free auraptene (AUR) with AUR concentration of (b) 7.5 and (c) 15 µg/mL, (d) PCL-PEG-PCL (TB), auraptene -loaded PCL-PEG-PCL (TB-AUR) with AUR concentration of (e) 7.5 and (f) 15 µg/mL, (g) PLA- PCL-PEG-PCL-PLA (PB) nanoparticles, auraptene-loaded PLA- PCL-PEG-PCL-PLA (PB-AUR) nano-particles with AUR concentration of (h) 7.5 and (i) 15 µg.mL. (**B**) Quantitative results of apoptotic effects evaluated by Annexin V/FITC assay.

Annexin-V/FITC test was carried out using flow cytometry technique as a quantitative test for study of early apoptotic, late apoptotic and necrotic cells. Figure 11 shows the quantitative results of Annexin V-FITC assay. Viable cells population in blank TB and PB NPs treated groups were near to control group. In 7.5 µg.mL^−1^ treatment groups, population of all early and late apoptotic cells in AUR, AUR loaded TB and AUR loaded PB groups were 22.5, 11 and 21% respectively. By increasing the AUR concentration to 15 µg.mL^−1^ in AUR, AUR loaded TB and AUR loaded PB treatment groups’ population of all early and late apoptotic cells were increased to 42, 21.5 and 64%, respectively. Therefore, highest population of apoptotic cells among all treatments over 72 h was observed in AUR loaded PB (15 µg.mL^−1^) group.

**Figure 12:**
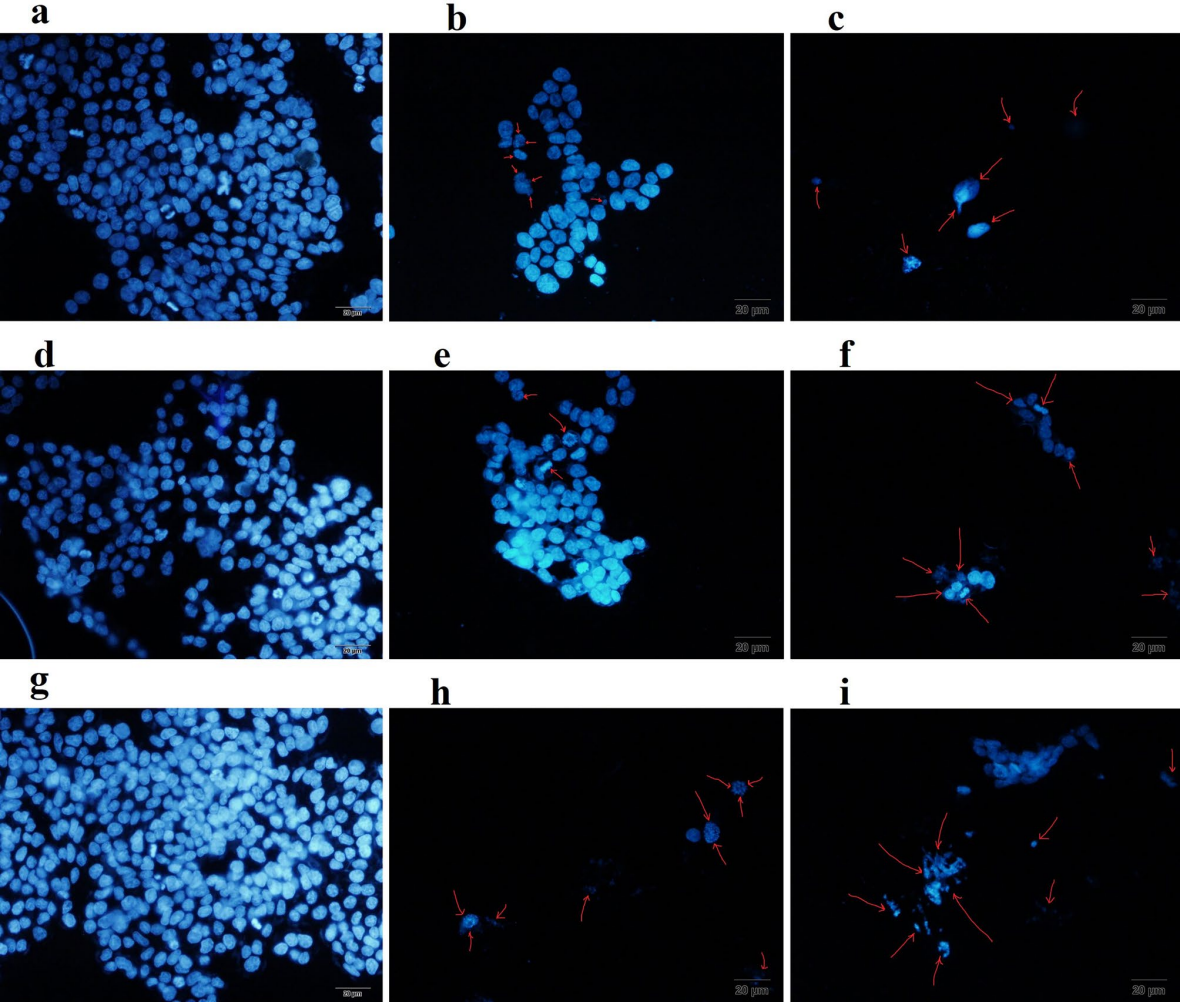
Microscopic images of DAPI stained HT-29 cells following 72 h of exposure to untreated cells as (**a**) control, free auraptene (AUR) with AUR concentration of (**b**) 7.5 and (**c**) 15 µg/mL, (**d**) PCL-PEG-PCL nanoparticles (TB), auraptene -loaded PCL-PEG-PCL nanoparticles (TB-AUR) with AUR concentration of (**e**) 7.5 and (**f)** 15 µg.mL^−1^, (**g**) PLA- PCL-PEG-PCL-PLA (PB) nanoparticles, auraptene -loaded PLA- PCL-PEG-PCL-PLA (PB-AUR) nanoparticles with AUR concentration of (**h**) 7.5 and, (**i**) 15 µg.mL^−1^.

DAPI staining was utilized as a complementary assay for evaluation of apoptosis in HT-29 cells. HT-29 cells were treated with AUR, AUR loaded TB, and AUR loaded PB with two concentration (7.5 and 15 µg.mL^−1^) as well as blank TB and PB (1000 µg.mL^−1^) NPs. HT-29 cells which were incubated with blank PB and TB NPs for 72 h showed normal nucleuses like control group (Fig. 12d,g). Apoptotic bodies and fragmented DNAs as a particular sign of apoptotic cells were observed in AUR, AUR loaded TB, and AUR loaded PB treatment groups (AUR concentration of 7.5 and 15 µg.mL^−1^) and were presented in the images by arrows (Fig. 12b–i). HT-29 cells population in treatment groups containing 15 µg.mL^−1^ of AUR were markedly decreased. The most apoptotic cells were observed in HT-29 cells treated with AUR loaded PB group with AUR concentration of 15 µg.mL^−1^.

### Cell cycle arrest

**Figure 13:**
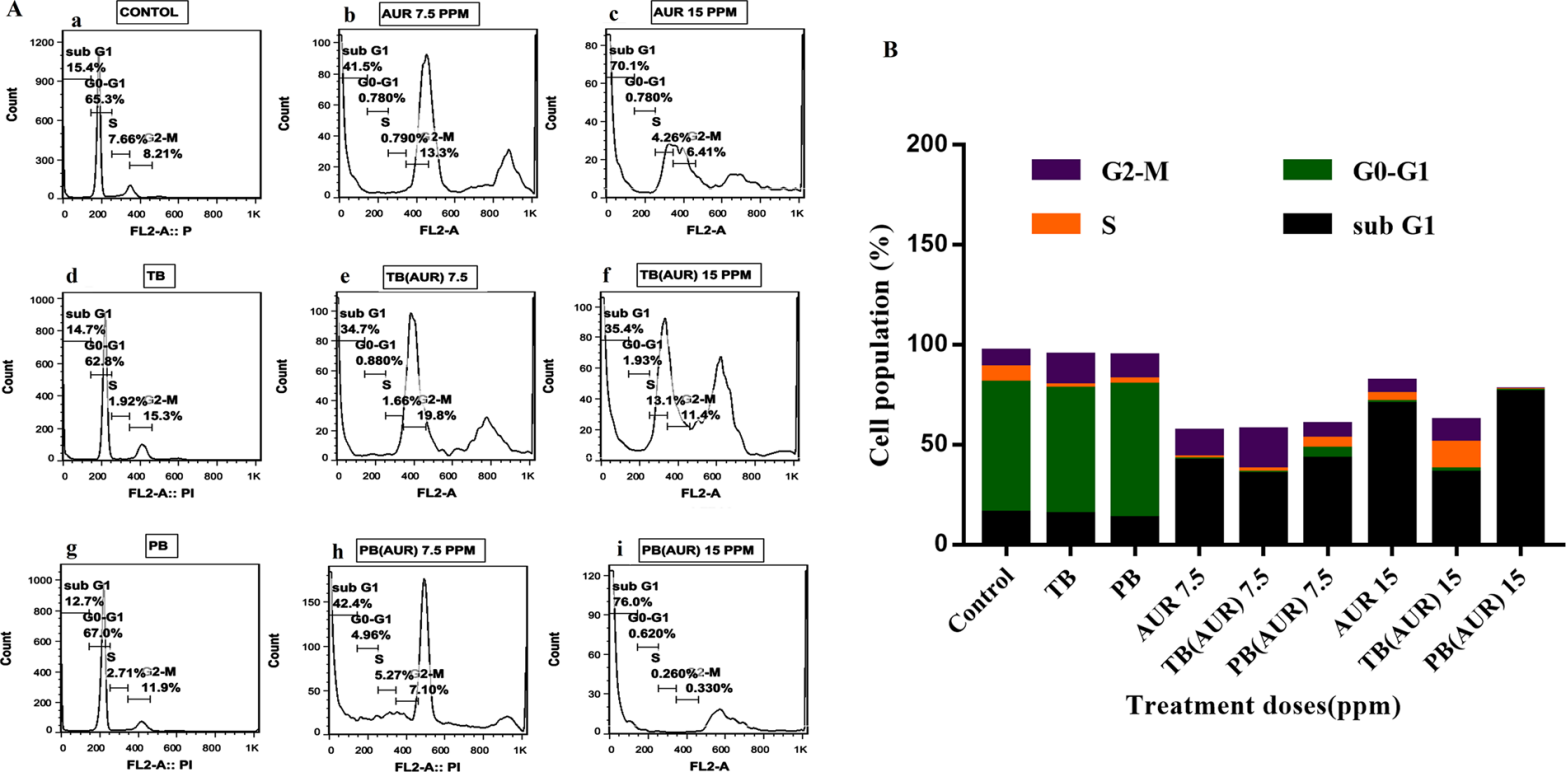
(**A**) Cell cycle analysis was performed by staining the DNA content of the cell followed by flow cytometry, percentage of cells in G0/G1, S or G2/M phase is indicated at (a) untreated cells as control, free auraptene (AUR) with AUR concentration of (b) 7.5 and (c) 15 µg/mL, (d) PCL-PEG-PCL (TB) nanoparticles, auraptene-loaded PCL-PEG-PCL nanoparticles (TB-AUR) with AUR concentration of (e) 7.5 and (f) 15 µg/mL, (g) PLA-PCL-PEG-PCL-PLA (PB) nanoparticles, auraptene -loaded PLA-PCL-PEG-PCL-PLA (PB-AUR) nanoparticles with AUR concentration of (h) 7.5 and, (i) 15 µg.mL^−1^. (**B**) Quantitative results of cell cycle arrest and distribution.

Cell cycle graphs showed that, cell cycle patterns of HT-29 cells which were treated with blank TB (Fig. 13d) and PB (Fig. 13g) NPs were not changed compared to untreated cells. Population of sub G1 region arrested cells was increased in AUR loaded PB, AUR loaded TB and free AUR at both concentrations of AUR (7.5 and 15 µg.mL^−1^).

Population of sub G1 region arrested cells in free AUR, AUR loaded TB and AUR loaded PB groups at AUR concentrations of 15 µg.mL^−1^ were 70, 35 and 76%, respectively. AUR loaded PB at AUR concentration of 15 µg.mL^−1^ showed highest population of sub G1 region arrested cells (~76%) among all treatment groups (Fig. 13i).

### Bax/Bcl-2 genes expression ratio

**Figure 14:**
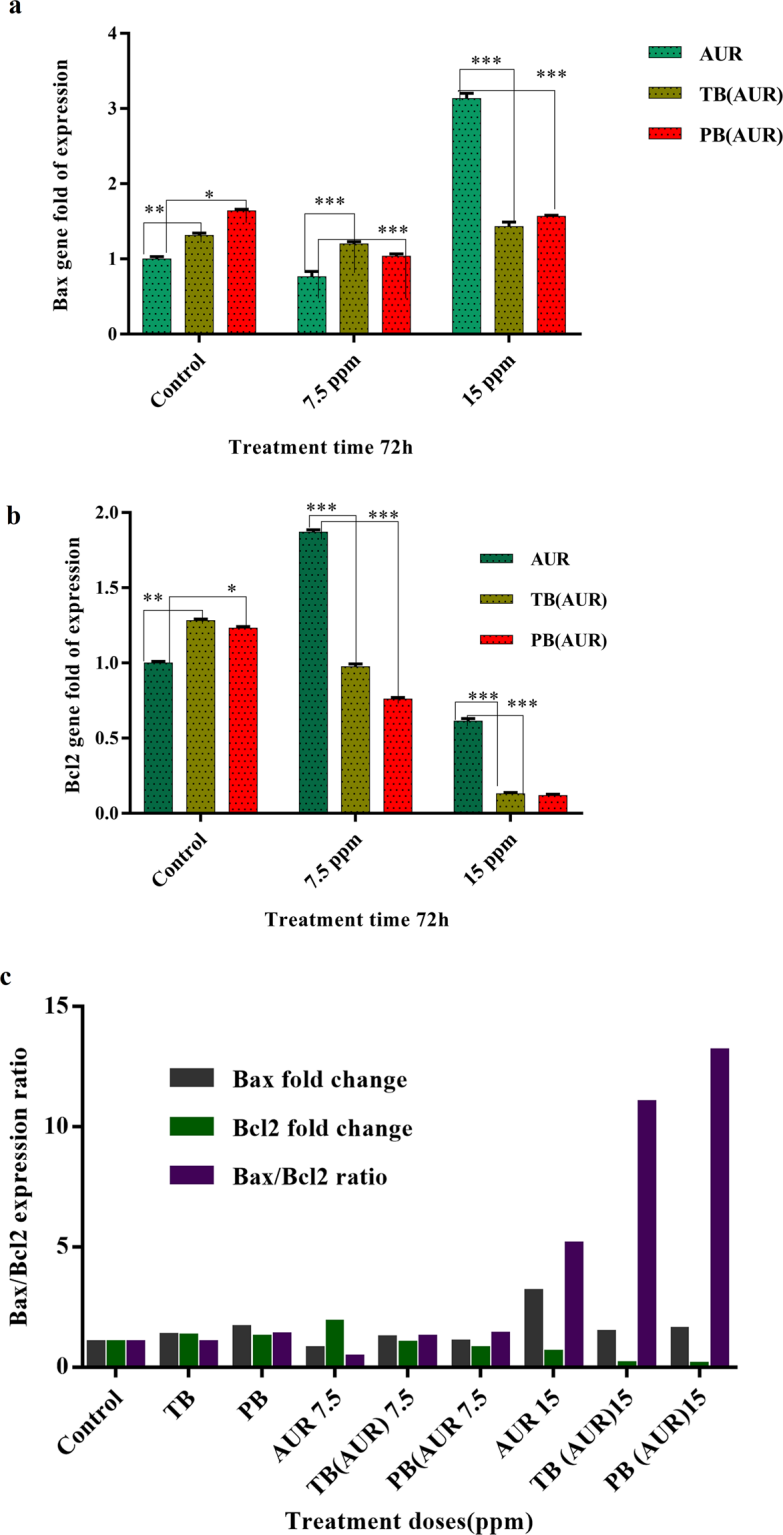
The impact of free auraptene (AUR), auraptene-loaded PCL-PEG-PCL (TB-AUR) and auraptene-loaded PLA-PCL-PEG-PCL-PLA (PB-AUR) nanoparticles with AUR concentration of 7.5 and 15 µg/mL on (**a**) Bax and (**b**) bcl2 gene expression and (**c**) Bax/bcl2 ratio results after 72 h of treatment. The results were expressed as mean ± SD from three independent experiments. *p < 0.05, **p < 0.01 and ***p < 0.01.

RT-PCR results indicated that expression of both Bax and Bcl2 genes in HT-29 cells treated with blank TB and PB NPs (1000 µg.mL^−1^) were similar to control group (untreated HT-29 cells). In 7.5 µg.mL^−1^ of AUR, AUR loaded TB and AUR loaded PB treatments any significant expression change weren’t observed compared to control group (Fig. 14). Remarkable increase in Bax/Bcl2 expression ratio was observed in 15 µg.mL^−1^ dose of free AUR, AUR loaded TB and AUR loaded PB treatment groups were observed. Highest Bax/Bcl2 gene expression ratio (around 13) was achieved in 15 µg.mL^−1^ AUR loaded PB treatment group. All treatment groups are statistically significant (p value < 0.05).

## Discussion

In this study we synthesized both triblock (PCL–PEG–PCL) (TB) and pentablock (PLA-PCL-PEG-PLA) (PB) copolymers to prepare appropriate NPs for encapsulation of AUR in a comparative manner. Poor solubility of AUR leads to limited bio distribution and bioavailability. Therefore, this study along with investigating differences of NPs prepared from TB and PB copolymers, attempted to increase AUR bio distribution by proper nano-formulations. Some polymer characteristics including molecular weight, hydrophobicity, and block arrangement affected particle size and drug release profile. As earlier reports suggested, by modulating hydrophobic/hydrophilic segments of copolymers, crystallinity and degradation of copolymers can be changed^26–29^. To change the crystallinity of PCL-triblock copolymer (PCL–PEG–PCL), PLA block was introduced to synthesis pentablock copolymer^30^. In this study we found that polymer structure and particle preparing method affect NPs size, PDI and surface charge. NPs characterization results indicated that NPs prepared from PB copolymer and USH method showed lower size than TB copolymer. Polymer structure and drug to polymer ratio affected drug loading capacity and encapsulation efficiency which AUR encapsulation efficiency and loading capacity in PB NPs showed 25% and 10% increase compared to TB NPs in type I formulation. In formulation type II and III by increasing of polymer to drug ratio the differences between TB and PB were not remarkable in terms of entrapment efficiency and loading capacity. As other studies reported that drug loading capacity and entrapment efficiency of NPs are mostly depended on copolymer compositions in which drug entrapment can be enhanced by increasing the molecular weight of lipophilic part of copolymers^22,24^.

NPs synthesis methods are divided into low-energy and high-energy methods. In high-energy methods, NPs obtain in short time and the size of NPs can be reduced to a desirable level^31^. In the present study, NPs were synthesized by two kind of high-energy methods (Ultrasonic Homogenization (USH) and Rotor Stator Homogenization (RSH)). DLS determines the hydrodynamic size, PDI (polydispersity index) and surface charge (zeta potential) of NPs. We observed differences in size, PDI, and zeta potential of NPs prepared with different methods. NPs prepared by USH method indicated acceptable negative surface charge, lower PDI values and smaller particle size than RSH methods. Lower PDI values indicate the uniformity of size in the synthesized particles^32,33^. The PDI values of both TB and PB NPs which prepared by USH method were below 0.29 but in RSH method ranged from 0.68–0.8. Therefore, due to the lower PDI value of USH method, NPs prepared by this method were chosen for cellular studies. In the SEM studies several particles size determined for both TB and PB NPs and all particles size were in the range of 10 nm to 50 nm. Particles measurements which achieved from SEM images were approximately 5 to 10 times smaller than the hydrodynamic size obtained by DLS technique. These results were in agreement with previously reported works^34^. Our findings revealed that utilizing ultrasound homogenization for preparing NPs was superior in terms of particle size and entrapment efficiency compared to classical rotor stator dispersion as another study reported that ultrasound homogenization technique was more effective in producing nano suspensions^35^. Zeta potential is a remarkable physicochemical characteristic that affects the stability of nano-suspensions which higher positive or negative zeta potential induces higher repulsive forces^36^. In our study surface charge of blank TB and PB NPs prepared by USH method were −9.26 and −17.5 mV, respectively which these values for AUR loaded TB and PB were −16.4 and −15 mV, respectively. All zeta values were in desirable range. Based on other reports in order to reach a combined high electrostatic and steric stability minimum acceptable range of zeta potential is ±20 mV^36^.

In physiological point of view, higher charge of particles, whether positive or negative, leads to NPs absorption by liver phagocytes which leads to particles disposition from the body^37,38^.

Small size of NPs causes to more stability of the suspension, deeper penetration into the tissues, and higher intracellular uptake and^39,40^.

Release study results revealed that both AUR loaded TB and PB NPs exhibited sustained release manner with slight lower mean amount of AUR release from TB NPs compared to PB NPs. We found that the differences between release profiles of each of NPs depended on physiochemical characteristics such as NPs sensitivity to temperature, solubility, crystallinity, molecular weight and rate of degradation. Previous studies reported that these synthesized copolymers (PCL-PEG-PCL and PLA-PCL-PEG-PCL-PLA) are thermosensitive hydrogels and pentablock copolymer indicates better solubility, lower crystallinity and faster degradation compared to triblock copolymer due to the presence of PLA blocks which directly affected drug release profile^22,24,41^.

Rhodamine b loaded TB and PB NPs were used to evaluate the percentage of NPs cellular uptake. The result of this experiment showed that both TB and PB NPs indicated high ability to enter the cells. Cellular uptake of PB NPs was higher than TB NPs due to the smaller size compared to TB NPs. TB NPs uptake at 0.5 h was higher than PB NPs maybe due to the lower negative charge of TB (−9.25) compared to PB (−17.5) leads to more intracellular uptake at first but throughout the time size of NPs played decisive role in intracellular absorption^33^.

In our study, the IC_50_ value of free AUR, AUR loaded TB and AUR loaded PB on HT-29 cells which were obtained by MTT assay were 13.05, 31.9 and 10.1 µg.ml^−1^, respectively. Based on the results achieved from MTT assay, in terms of optimal dose and exposure time, the experiments were carried out in two 15 µg.mL^−1^ (IC_50_ of AUR) and 7.5 µg.mL^−1^ (sub IC_50_ dose) for 72 h. After 48 h exposure of HT-29 cells to mentioned treatment groups, cell mortality rate in free AUR was higher than AUR loaded TB and AUR loaded PB due to the slow release of drug from micelles^22^. But over 72 h incubation, AUR loaded PB showed superior cell cytotoxic effects compared to AUR loaded TB and free AUR.

Previous studies on the effects of auraptene on various malignant and nonmalignant cells revealed that auraptene functions in a dose and time dependent manner. It has also been reported that exposure of 20μm of AUR to normal oral epithelial cells had no deleterious effects^8^. AUR lethal dose on snu-1 cells has been reported 25μm, while it had no harmful effects on normal hek-293 cells which can be concluded that, AUR has a high potential for treating gastric cancer without damaging normal cells^42^.

AUR which has been used in a non-lethal dose in combination with chemotherapeutic agents (fluorouracil, paclitaxel and cisplatin) has increased the ability of these chemotherapeutic drugs in induction of apoptosis in cancer cells^43^. A study which investigated the effects of AUR on MCF 7 breast cancer cells, it has been reported that, AUR at 20 and 50 μM concentrations significantly reduced cell growth to 26% and 49%, respectively^44^. This finding was in consistent with our results were obtained from growth inhibition of HT-29 cells by auraptene.

Apoptosis is series of molecular sequences which can be induced in different tumor cells by many chemotherapeutic drugs and approved natural substances^45^. Apoptosis induction by AUR on various cancer cell lines and weakening of tumor progression in some animal models have been reported previously^43,45,46^. Several studies have also revealed that AUR induces apoptosis in colon carcinoma cells, including, LNCAP (sensitive to androgens), DU145 (insensitive to androgens), PC3, and SNU-1, by activating P53 and inhibiting mTOR pathways^42,47^.

In order to evaluate the improved therapeutic index of encapsulated AUR, apoptotic assays including Annexin V-FITC, DAPI staining and cell cycle tests were performed. Sub-G1 peak in cell cycle analysis of treatment groups containing 7.5 and 15 µg.ml^−1^ concentration of AUR was observed. In a study by Afshari *et al*., cytotoxic effects of AUR on human malignant glioblastoma cells was studied. They reported sub-G1 cell cycle arrest which is in accordance with our results^48^. In the results of Annexin V/ FITC test, apoptotic population of HT-29 cells treated with free AUR and AUR loaded PB was increased from 41.35% to 64.8% with the same dose of AUR (15 µg.ml^−1^), respectively. Consequently, encapsulation of AUR improved potential of apoptosis induction in HT-29 cancer cells about 25%. AUR loaded PB showed the maximum population of early and late apoptotic cells compared to free AUR and AUR loaded TB (Fig. 9e,f). Therefore, we concluded that AUR loaded PB can function as a long lasting most efficient treatment group.

Apoptotic bodies and fragmented DNAs as a particular character of apoptotic cells were also observed by florescent microscope. In the results of DAPI staining, apoptosis induction was observed in both 7.5 and 15 µg.ml^−1^ of AUR, AUR loaded TB and AUR loaded PB. Higher population of apoptotic HT-29 cells were observed in AUR loaded PB containing 15 µg.mL^−1^ AUR (Fig. 10i). Untreated HT-29 cells (Fig. 10a) and cells had been treated with blank TB (Fig. 10d) and PB (Fig. 10g) NPs indicated nucleuses with normal appearance and morphology which were in accordance with findings had been obtained by Annexin-V test.

Pro-apoptotic Bax and anti-apoptotic Bcl2 genes are members of Bcl2 family which play important role in mitochondrial apoptosis^49–51^. Bax/Bcl2 expression ratio is important at clinical level as a remarkable prognostic and diagnostic marker in different diseases including cancers. In this study real time PCR array carried out to analyze changes in expression of Bax and Bcl2 genes by free AUR, AUR loaded TB and AUR loaded PB treatment groups. It is noteworthy that based on all assays and finally real time PCR data, TB and PB copolymers were completely safe and biocompatible. Real time PCR results showed no significant change in Bax/Bcl2 genes ratio in sub IC50 dose (7.5 µg.mL^−1^) of free AUR, AUR loaded TB and AUR loaded PB treatment groups compared to control group which can be explained by dose dependency of auraptene^47^. In a study which was done by Kawabata *et al*., they studied auraptene effects on translation of MMP-7 in HT-29 colon carcinoma cells, they found that auraptene remarkably inhibited the production of proMMP-7 protein, without affecting its mRNA expression level^52^. They also investigated the effects of 25 µM concentration of AUR (7.5 µg. mL^−1^) on the mRNA expression of Bcl-xL protein (as an anti-apoptotic member of bcl2 family). They reported that auraptene markedly inhibited the translation of that protein, whereas no significant changes in the mRNA expression level was observed^52^. This results was in accordance with our findings. In addition, auraptene is a bioactive antioxidant coumarin with valuable pharmacological properties that indicates anti-inflammatory effects in lower concentrations^8^. The anti-inflammatory activity of AUR can active various molecular mechanisms^52,53^.

Our Real time PCR results indicated that Bax /Bcl2 expression ratio as an apoptosis predicting criterion, in free AUR, AUR loaded TB and AUR loaded PB at IC_50_ dose of AUR (15 µg.mL^−1^) increased 6, 9 and 13 times compared to control, respectively (*p*-value < 0.05). Lee and colleagues in 2017, reported AUR induced apoptosis through activating caspase 3 and 9 along with down regulation of anti-apoptotic Bcl2 and Mcl1, and up regulation of pro apoptotic Bax proteins in prostate cancer cells^47^.Our results revealed that AUR loaded PB at 15 µg.mL^−1^ concentration can initiate apoptosis by modulation of Bax and Bcl2 genes expression in colon carcinoma HT-29 cells more efficiently than AUR loaded TB and free AUR.

## Conclusion

AUR as one of the most abundant geranyloxy coumarins in the nature has many valuable pharmaceutical properties such as high anti-cancer, antibacterial anti-fungal, antioxidant and anti-inflammatory effects. Low solubility of this compound in aqueous solutions leads to low bio-distribution and poor delivery to targeted sites which has limited its pharmaceutical applications. This study aimed to design an efficient tool for delivery of AUR and investigation of impact of encapsulated form of this natural substance on inhibition of proliferation in colon cancer cells. In this study nano-encapsulated AUR with triblock PCL-PEG-PCL and penta-block PLA-PCL-PEG-PCL-PLA biodegradable and biocompatible FDA approved copolymers were prepared. NPs were desirable in terms of size, charge, shape, topography, drug intake and intracellular absorption. Our results confirmed the improvement of AUR anticancer properties in colon cancer cells. Our findings suggest that AUR micelles especially penta-block nano formulation has improved therapeutic indexes of AUR. Because of intrinsic chemo preventive activity, AUR loaded micelles can be considered as a promising strategy to develop effective, safe and sustain therapies against colon cancer.

## Experimental

### Materials

Poly ethylene glycol (PEG: 1 k da), stannous octoate, ε-caprolactone, D, L-lactide and polyvinyl alcohol (Mw 89000-98000) (PVA) were bought from Sigma-Aldrich (st. Louis, mo., USA). 3-(4,5-dimethylthiazol-2-yl)-2,5-diphenyltetrazolium bromide (MTT), and all other biological materials including propidium iodide (PI), ribonuclease A, and rhodamine B were purchased from Sigma-Aldrich co. Trypsin, fetal bovine serum (FBS), and Roswell park memorial institute 1640 growth medium (RPMI) were obtained from GIBCO BRL life technologies.

Solvents and all other chemicals were obtained from Merck co and used as received. Deionized water utilized in all assays was purified with a milli-q water system.

Auraptene (AUR) a sesquiterpene coumarin from Ferula szowitsiana, as an anticancer agent, was extracted and purified (>95% purification) by Dr Iranshahi in Pharmacognosy Lab of Mashhad University of Medical Sciences.

### Polymers synthesis

TB and PB copolymers were synthesized by ring opening polymerization method. In the first step, triblock (TB) copolymer PCL–PEG–PCL (Fig. 1) was synthesized by ring opening polymerization of ε-caprolactone on two hydroxyl ends of PEG (Mw1000) as initiator, and stannous octoate was added as catalyst. For synthesis of triblock copolymer, PEG (Mw1000) was dried under vacuum for three hours before copolymerization. Predetermined amounts of peg (7.5 g), ε-caprolactone (15 ml), and stannous octoate (1 wt %) were poured in polymerization tube and exposed to nitrogen flow for 30 min. Then the reaction was continued for 24 h at 130 °C and obtained product was dissolved in methylene chloride and precipitated with chilled petroleum ether to eliminate none-reacted monomers. The precipitated polymer was filtered and vacuum-dried for 24 h. After purification a calculated quantity of PCL-PEG-PCL was utilized as macroinitiator for synthesis of D, L-lactide- containing pentablock copolymer (PLA-PCL-PEG-PCL-PLA). For synthesis of pentablock copolymer, triblock copolymer (5 g) and L-lactide (10 ml) monomer were poured in the round-bottom flask, and stannous octoate (1 wt %) was utilized as a catalyst. Then, the flask was exposed to nitrogen, reaction was continued for 24 h at 130 °C. Finally obtained product was dissolved in methylene chloride and precipitated with cold petroleum ether, sediment was vacuum dried for 24 h.

### Characterization of polymers

#### Hydrogen nuclear magnetic resonance (HNMR)

Synthesized TB and PB copolymers were characterized by Hydrogen nuclear magnetic resonance (^1^H NMR) spectroscopy using Bruker spectra spin 400 MHz (Germany).

#### Gel permeation chromatography

Gel permeation chromatography (GPC Agilent 110) was used to measure the molecular weight (M_W_) of PCL-PEG-PCL and PLA-PCL-PEG-PCL-PLA copolymers. Tetrahydrofuran (THF) at a concentration of 1 mg/mL used to dissolve the samples. THF was eluted at a rate of 1 min/mL through PL gel mixd C-10 μm column and a linear column. The external and column was kept at 30 °C and standard Polystyrene used to calibrate the GPC.

#### Fourier transforms infrared (ftir) spectroscopy

The chemical structures of the synthesized TB and PB copolymers as well as AUR loaded TB and PB NPs were studied by FTIR Spectroscopy. The FTIR spectra were recorded on Bruker tensor 270 spectrometer.

### Preparation of nanoparticles

NPs were prepared by RSH and USH methods. At first predetermined amount of polymers were dissolved in 5 ml of dimethyl sulfoxide (DMSO). Then polymer solution was added dropwise to poly vinyl alcohol (PVA) (0.25% Wt%) solution while homogenizing with rotor stator homogenizer (RSH) at 20000 rpm or ultrasonic homogenizer (USH) probe with 90% homogenization power of 400 W. The prepared NPs suspensions were centrifuged three times to remove DMSO, re-dispersed in deionized water and saved at 25 °C for size and morphology studies.

### Auraptene encapsulation studies

For preparing of AUR loaded nanoparticles, at first AUR and polymers (both TB and PB) were dissolved in DMSO and stirred for 24 h. Coupled with drug loading process in order to evaluate AUR entrapment ability of copolymers, TB and PB loaded nanopaeticles were prepared in three different ratio of polymer to drug (2:1, 5:1 and 10:1) using 0.25% Wt% of PVA solution and homogenized with either ultrasonic homogenizer or rotor stator homogenizer. The nanoparticle suspensions were centrifuged at 12000 rpm for 1 hour and wash several times with deionized water to eliminate the unloaded AUR then the suspension was separated by centrifugation and diluted with deionized water. Then UV–vis spectrophotometer (UV160-shimadzo −Japan) was applied to measure the amount of AUR loading at ƛmax = 320 nm. The encapsulation efficiency and loading capacity of AUR loaded TB and PB NPs were calculated by following equations:

$$\begin{array}{rcl}{\rm{encapsulation}}\,{\rm{efficiency}}( \% ) & = & \frac{{\rm{Mass}}\,{\rm{of}}\,{\rm{initial}}\,{\rm{drug}}-{\rm{Mass}}\,{\rm{of}}\,{\rm{free}}\,{\rm{drug}}}{{\rm{mass}}\,{\rm{of}}\,{\rm{initial}}\,{\rm{drug}}}\times 100\\ {\rm{loading}}\,{\rm{capacity}}( \% ) & = & \frac{{\rm{Mass}}\,{\rm{of}}\,{\rm{total}}\,{\rm{entrapped}}\,{\rm{drug}}\,}{{\rm{Mass}}\,{\rm{of}}\,{\rm{polymer}}}\times 100\end{array}$$

### NPs characterization

#### Dynamic light scattering

In order to measure the average diameter size and zeta-potential of NPs, the laser scattering technique was used at 25 °C using a zeta sizer nanoZs90, Malvern instruments, Malvern, UK.

#### Scanning electron microscopy

Surface topography and dimensions of NPs were observed by field emission scanning electron microscope energy dispersive X-ray (SEM-EDX, Tuscan Mira3, Czech). The specimens were overspread on a sheet and coated with gold. The particles size was achieved by measuring the diameters of at least 100 particles revealed through SEM, utilizing image analysis software (Image-Pro plus 4.5; Media Cybernetics, Silver Spring, MD).

### Drug release profile

In order to study AUR release from both AUR loaded TB and AUR loaded PB nano-suspensions, at first both formulations were prepared in polymer to drug ratio of 2 to 1. 1 ml of both formulations containing 150 µg AUR were poured in 5 ml phosphate buffer solutions containing 0.1% tween 80 (different pH values: pH 5.4 and 7.4) and shook at a rate of 100 rpm in an incubator at 37 °C. At predetermined time intervals from 0.5 h up to 120 h, samples were centrifuged and supernatants were collected and replaced with equal volume of fresh buffer. Released AUR were measured at 320 nm using a UV-visible spectrophotometer. The detected absorbance of AUR was converted to their concentration according to the calibration curve of AUR in the same buffer. Then, the relative percentage of the released AUR were calculated as a function of incubation time and based on the amount of the drug existed in the nanoparticles. Percent of drug released from nanocomposites was calculated by the following equation:

$$Released\,drug( \% )=\frac{Mass\,of\,drug\,in\,released\,medium\,}{Mass\,of\,drug\,loaded\,to\,nanoparticles}\times 100$$

### Cellular uptake assays

Intracellular uptake was performed to examine the ability of the NPs incorporate into the cell. In order to study of nanoparticles cellular uptake, rhodamine b dye was used as a fluorescent tracer to prepare fluorescent NPs. Rhodamine b were loaded with rhodamine to polymer ratio of 1 to 200. All processes were carried out under dark condition according to drug loading protocol mentioned above. Afterward the rhodamine b labeled NPs were collected by centrifugation and washed three times to eliminate the unloaded rhodamine b. HT-29 cells were cultured in 6-well plates at density of 2 × 10^5^ cells per well and after reaching to confluence of over 70% were incubated with rhodamine b labeled NPs for 0.5, 1.5, 3, 24 hours. Untreated cells were considered as control group, and rest of groups were compared against control.

### Epifluorescence microscopy

To qualitative study of nanoparticles intracellular uptake, at first HT-29 cells were cultured in slid chamber at density of 5 × 10^4^ cells per well and after reaching the confluence of over 70% were treated with rhodamine b labelled NPs for 3 hours.

HT-29 cells were treated by rhodamine b loaded NPs also stained with DAPI. Enhanced depth of focus (EDOF) microscopic techniques were utilised according to Ghafariyan *et al*.^54^. The stained samples were viewed through a Nikon E1000M (Nikon, Tokyo, Japan) research fluorescence microscope equipped with the Planapo apochromatic objectives (Nikon, Tokyo, Japan). The best fluorescence excitation observed when mirror cube units for 480–510 nm and 510–550 nm were used. Consecutive image series from successive focal plates (with 1 μm increment per focal step for 60x objective) was acquired by means of an Evolution MP cooled CCD (Media Cybernetics, USA) according to a methodology introduced by Dadpour *et al*.^55^. Digital images were taken by TIFF format using RGB mode (12 bits per each channel) with the resolution of 2560 × 1920 pixels.

### Image analysis

Acquired consecutive serial image were imported to Image J 1.37 software (freely available from http://rsb.info.nih/ij/) to produce a Z stack based on method provided by Peighambardoust *et al*.^56^. Then, image level was adjusted in entire of images stack. Subsequently, image sharpness was improved by application the Unsharp mask filter at the moderate range (Gaussian radius = 5 and Mask weight = 0.5). The enhanced Z-stack was then processed for automatically improving depth of focus, using Z-function plug in^57^. Outputs from Z-stack were trimmed to proper size and scale bars were then added to final images based on optical magnification. Furthermore, a three dimensional image for each image stack was produced after Z stack composition for better positional determination of the nanoparticle clamps in the plant tissue exploiting image J 3D viewer plug in.

Intracellular uptake of nano particles was further confirmed by fluorescence microscopy (qualitative method). HT-29 Cells were grown on slide chambers after 24 h, cells were treated with Rhodamine B labeled NPs. After incubation for 0.5, 1.5 and 3 h, the cells were rinsed with PBS and Rhodamine B labelled nano particles uptake were observed using a fluorescence microscope (Olympus microscope Bh2- FCA, Japan).

### Cell viability assay

Relative cytotoxicity’s of all treatment groups including TB and PB blank NPs, free AUR and AUR loaded TB and PB NPs were evaluated on HT-29 colon cancer cells by MTT assay for 24, 48, and 72 h according to the following procedure.

In brief, HT-29 cells (obtained from Pasteur institute of Iran, Tehran, Iran) were cultured in 96-well plates (1 × 10^4^ cells per well) and incubated with RPMI 1640 medium containing 10% FBS, 1% penicillin/streptomycin at 37 °C atmosphere with 5% CO_2_ for 24 h. Cells were treated with medium containing blank NPs and AUR loaded PB and TB NPs as well as free AUR with various concentrations of the AUR. Cells without any treatment were utilized as negative control. In order to cell viability evaluation by MTT assay method after 24, 48, and 72 h incubation, HT-29 cells media were depleted and wells were rinsed twice with sterile PBS solution. 50 μl MTT solution (2 mg.mL^−1^) was subjoined to each well and incubated for 4 h. The unreacted MTT containing medium was carefully removed from each well, and 150 μl of DMSO was added to each well to solubilize formed blue formazan crystals. Absorbance of all wells were measured by an Elisa reader instrument (Biotek) at 570 nm with a reference wavelength of 630 nm, then based on obtained ODs cell viability values were calculated. All assays were performed in triplicate.

### Apoptosis assays

#### Flow cytometry annexin-V/PI apoptosis assay

To evaluate apoptosis, HT-29 cells were seeded in to six-well plates at a density of 5.0 × 10^5^ cells per well in the culture medium. After 24 h, cell culture mediums were replaced with fresh medium and treated with blank TB and PB NPs, free AUR and AUR loaded TB and PB NPs and incubated for 72 h. The drug dosage of the free drug, AUR loaded TB and AUR loaded PB was at 7.5 and 15 µg/mL (the IC50 and sub IC50 values). Then the cells were trypsinized, collected and centrifuged to remove the supernatant. The collected cells were rinsed one more time with chilled PBS to eliminate trypsin completely. Then 5 × 10^5^ number of cells were suspended in 200 µL Annexin binding Buffer. To stain apoptotic cells, a FITC/Annexin-V apoptosis detection kit was used based on the manufacture’s protocol, which is described as following: 100 μl of cells suspension, 5 μl FITC/Annexin-V and 5 μl PI (propidium iodide, provided in kit) were mixed, then cells were incubated for 15 min and finally 400 μl of binding buffer were added to each suspension, BD Facscalibur flow cytometry was used to analyze apoptotic cells.

#### DAPI staining method

DAPI staining was performed by the following protocol for observation of apoptotic cells after treatment with blank TB and PB NPs, free AUR, AUR loaded TB and AUR loaded PB NPs. In brief, HT-29 cells were cultured in six-well plates (5 × 10^4^ cells per well) and incubated at 37 °C for 24 h. Then, the culture media was treated by fresh media containing blank TB and PB NPs, free AUR, AUR loaded TB and AUR loaded PB NPs in which the concentrations of the AUR was at 7.5 and 15 µg/mL (the IC50 and sub IC50 values). After 72 h, cells were rinsed three times with PBS (PH 7.4) to eliminate any free drug and NPs. Finally, the cells were fixed with 4 w t% paraformaldehyde for 15 min at room temperature. Then, the cells were rinsed three times with PBS and permeablized with Triton x-100 (0.1% w/v) for 5 min. The cells were rinsed again with PBS and stained with 300 ng/mL DAPI for 15 min. In the images of DAPI staining HT-29 cells in all treatment groups were enumerated and were compared to control group and population of dead cells were calculated. DNA condensation and fragmentation in apoptotic cells were observed under the lens of fluorescence microscope (Olympus microscope Bh2-rfca, Japan).

### Cell cycle analysis

For analyzing DNA content as an index of cell generating, reagents including DNA dye such as propidium iodide (PI) are used. In this study samples of untreated and treated HT-29 cells were evaluated for DNA content during cell cycle by flow cytometry technique. Cell cycle distribution was determined after proper gating of cell populations in FL-2-Area.

In a six-well plate, HT-29 cells with density of 5 × 10^5^ were cultured and incubated for 24 h. Cells were treated with blank TB and PB NPs, free AUR, AUR loaded TB and AUR loaded PB NPs. The drug concentration of the free AUR and AUR loaded NPs was the IC50 of AUR after 48 h. To validate the non-toxicity of the blank TB and PB Nano-particles on HT-29 cells, the cells were exposed to 1000 μg.ml^−1^ of NPs. Untreated cells were set as control. After 48 h of incubation, cells were rinsed with PBS, trypsinized and harvested, then fixed using ice cold 70% ethanol, and kept at 4 °C for 3 days. Then cells were centrifuged and washed twice with PBS to remove the ethanol. Then cells were incubated with RNAse solution at 37 °C for 45 min and finally were stained with PI solution for 10 min. Detection of the cell population in different stages of cell cycle was evaluated by the BD facscalibur.

### Real time PCR gene expression study

Effects of blank TB and PB NPs, free AUR, AUR loaded TB and AUR loaded PB NPs on Bax and Bcl-2 genes expression were studied by q RT-PCR method. Total RNA was extracted using an RNA extraction set (Biobasic Inc) based on Manufacturer’s protocols. The cDNA synthesis was carried out using revers aid cDNA synthesis (Thermo Fisher Scientific). The syber green master mix (E Bioscience) was employed for producing primers (summarized in Table 4) on a MIC PCR system. Real-time PCR data were analyzed by 2ddct method. Beta-actin was utilized as housekeeping reference gene.

**Table 4:** Forward (F) and reverse (R) primer sequences of Bax, bcl2 and β-actin genes.

Gene	Primer Sequence
Bax	Forward 5′-TTCTGACGGCAACTTCAACT (sense)−3′ 20 Reverse 5′-CAGCCCATGATGGTTCTGAT (antisense)-3′ 20
Bcl-2	Forward 5′-GGGAATCGATCTGGAAATCCTC-3′ (sense) 20 Reverse 5′-GGCAACGATCCCATCAATCT-3′ (antisense) 20
β-actin	Forward: 5′-GGTGAAGGTGACAGCAGT-3′ (sense) 18 Revers: 5′-TGGGGTGGCTTTTAGGAT-3′ (antisense) 18

### Statistical analysis

Statistical analyzes were performed in graph pad prism (version: 6) and EXCEL Microsoft software. Differences between groups were assessed with two-way analysis of variance (ANOVA). Statistically, P value < 0.05 was considered significant.

### Ethical approval

This study does not include any human or animal participants

### Informed consent (Optional)

For this type of study, formal consent is not required

### Supplementary information

Supplementary Materials.
