# Author Correction: Novel nano-vehicle for delivery and efficiency of anticancer auraptene against colon cancer cells

**DOI:** 10.1038/s41598-024-56785-w

**Published:** 2024-04-03

**Authors:** Nazila Jalilzadeh, Naser Samadi, Roya Salehi, Gholamreza Dehghan, Mehrdad Iranshahi, Mohammad Reza Dadpour, Hamed Hamishehkar

**Affiliations:** 1 Faculty of Natural Sciences, University of Tabriz, Tabriz, Iran; 2 Department of Biochemistry and Clinical Laboratories, Faculty of Medicine, Tabriz University of Medical Sciences, Tabriz, Iran; 3 Drug Applied Research Center, Tabriz University of Medical Sciences, Tabriz, Iran; 4 Department of Medical Nanotechnology, Faculty of Advanced Medical Sciences, Tabriz University of Medical Sciences, Tabriz, Iran; 5 Faculty of Pharmacy, Mashhad University of Medical Sciences, Mashhad, Iran; 6 Department of Horticulture, Faculty of Agriculture, University of Tabriz, Tabriz, Iran

Correction to: *Scientific Reports* 10.1038/s41598-020-58527-0, published online 31 January 2020

The original version of this Article contained errors.

As a result of an error in the figure assembly, the panels of Figure 12 were positioned incorrectly.

The original Figure 12 and its accompanying legend appear below.

**Figure 12:**
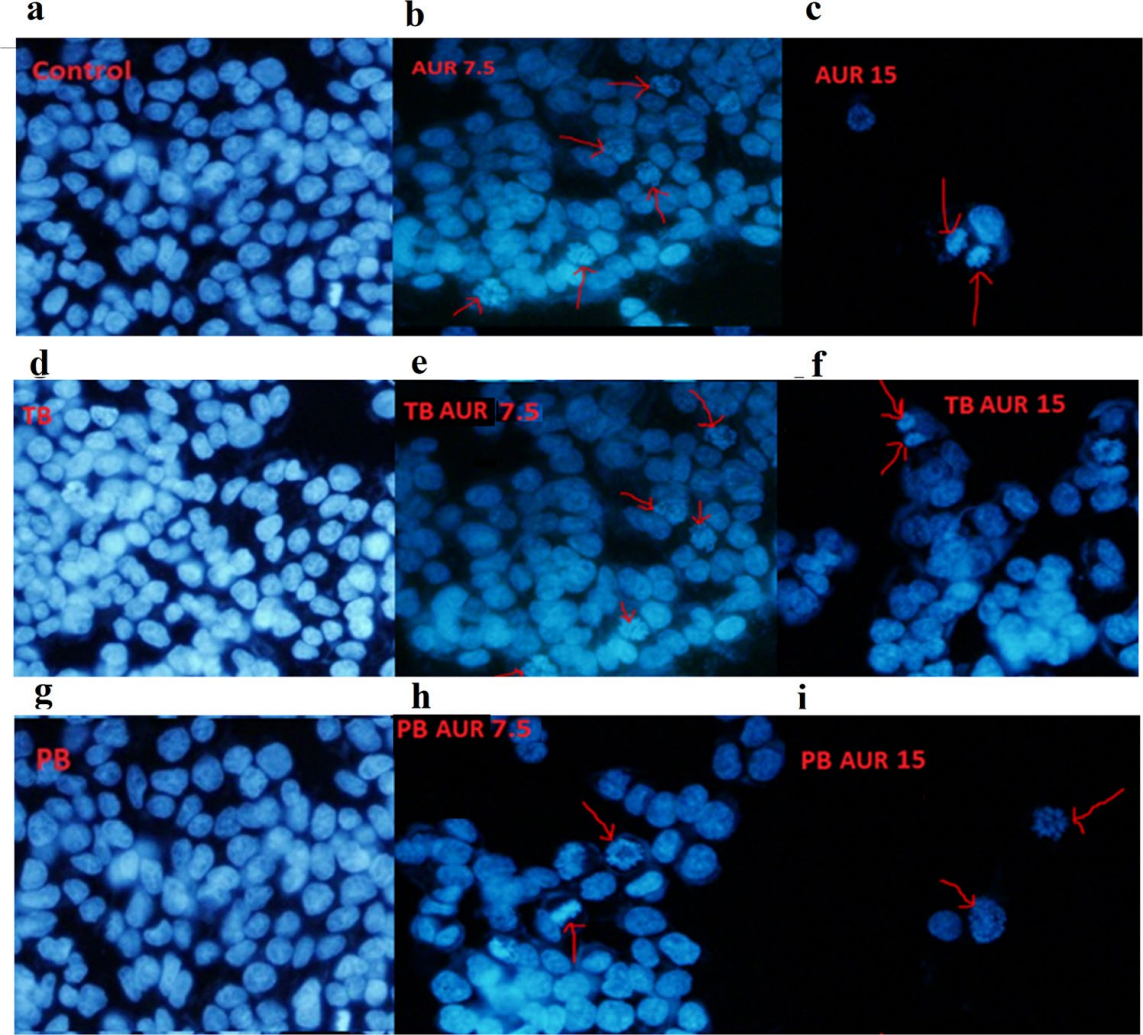
Microscopic images of DAPI stained MCF7 cells following 72 h of exposure to untreated cells as (**a**) control, free auraptene (AUR) with AUR concentration of (**b**) 7.5 and (**c**) 15 µg/mL, (**d**) PCL-PEG-PCL nanoparticles (TB), auraptene-loaded PCL-PEG-PCL nanoparticles (TB-AUR) with AUR concentration of (**e**) 7.5 and (**f)** 15 µg.mL^−1^, (**g**) PLA-PCL-PEG-PCL-PLA (PB) nanoparticles, auraptene-loaded PLA-PCL-PEG-PCL-PLA (PB-AUR) nanoparticles with AUR concentration of (**h**) 7.5 and, (**i**) 15 µg.mL^−1^.

In addition, the Article contained a repeated error in the legends of Figure 8, Figure 11 and Figure 12, where the ‘HT-29’ cell line was incorrectly given as ‘MCF7’.

As a result, the legend of Figure 8:

“Rhodamine b labelled PCL-PEG-PCL Triblock (TB) and PLA-PCL-PEG-PCL-PLA pentablock (PB) nanoparticles uptake by MCF7 cells after different exposure times of 0.5, 1, 2, 3 and 24 h assessed by flow-cytometry.”

now reads:

“Rhodamine b labelled PCL-PEG-PCL Triblock (TB) and PLA-PCL-PEG-PCL-PLA pentablock (PB) nanoparticles uptake by HT-29 cells after different exposure times of 0.5, 1, 2, 3 and 24 h assessed by flow-cytometry.”

The legend of Figure 11:

“Annexin V and PI staining was used to identify viable cells (annexin V−, PI−), early apoptotic cells (annexin V+, PI−), late apoptotic (annexin V+, PI+) and necrotic cells (annexin V−, PI+). (**A**) The apoptotic effects of cells, determined by flow cytometry after 12 h in MCF7 cells for (a) untreated cells as a control, free auraptene (AUR) with AUR concentration of (b) 7.5 and (c) 15 µg/mL, (d) PCL-PEG-PCL (TB), auraptene-loaded PCL-PEG-PCL (TB-AUR) with AUR concentration of (e) 7.5 and (f) 15 µg/mL, (g) PLA-PCL-PEG-PCL-PLA (PB) nanoparticles, auraptene-loaded PLA-PCL-PEG-PCL-PLA (PB-AUR) nano-particles with AUR concentration of (h) 7.5 and (i) 15 µg.mL. (**B**) Quantitative results of apoptotic effects evaluated by Annexin V/FITC assay.”

now reads:

“Annexin V and PI staining was used to identify viable cells (annexin V−, PI−), early apoptotic cells (annexin V+, PI−), late apoptotic (annexin V+, PI+) and necrotic cells (annexin V−, PI+). (**A**) The apoptotic effects of cells, determined by flow cytometry after 12 h in HT-29 cells for (a) untreated cells as a control, free auraptene (AUR) with AUR concentration of (b) 7.5 and (c) 15 µg/mL, (d) PCL-PEG-PCL (TB), auraptene-loaded PCL-PEG-PCL (TB-AUR) with AUR concentration of (e) 7.5 and (f) 15 µg/mL, (g) PLA-PCL-PEG-PCL-PLA (PB) nanoparticles, auraptene-loaded PLA-PCL-PEG-PCL-PLA (PB-AUR) nano-particles with AUR concentration of (h) 7.5 and (i) 15 µg.mL. (**B**) Quantitative results of apoptotic effects evaluated by Annexin V/FITC assay.”

The legend of Figure 12:

“Microscopic images of DAPI stained MCF7 cells following 72 h of exposure to untreated cells as (**a**) control, free auraptene (AUR) with AUR concentration of (**b**) 7.5 and (**c**) 15 µg/mL, (**d**) PCL-PEG-PCL nanoparticles (TB), auraptene-loaded PCL-PEG-PCL nanoparticles (TB-AUR) with AUR concentration of (**e**) 7.5 and (**f)** 15 µg.mL^−1^, (**g**) PLA-PCL-PEG-PCL-PLA (PB) nanoparticles, auraptene-loaded PLA-PCL-PEG-PCL-PLA (PB-AUR) nanoparticles with AUR concentration of (**h**) 7.5 and, (**i**) 15 µg.mL^−1^.”

now reads:

“Microscopic images of DAPI stained HT-29 cells following 72 h of exposure to untreated cells as (**a**) control, free auraptene (AUR) with AUR concentration of (**b**) 7.5 and (**c**) 15 µg/mL, (**d**) PCL-PEG-PCL nanoparticles (TB), auraptene-loaded PCL-PEG-PCL nanoparticles (TB-AUR) with AUR concentration of (**e**) 7.5 and (**f)** 15 µg.mL^−1^, (**g**) PLA-PCL-PEG-PCL-PLA (PB) nanoparticles, auraptene-loaded PLA-PCL-PEG-PCL-PLA (PB-AUR) nanoparticles with AUR concentration of (**h**) 7.5 and, (**i**) 15 µg.mL^−1^.”

The original Article has been corrected.

