# Supplementary material for: Novel nano-vehicle for delivery and efficiency of anticancer auraptene against colon cancer cells
Source: Sci Rep. 2020 Jan 31;10:1606. doi: 10.1038/s41598-020-58527-0 (PMC6994674; doi:10.1038/s41598-020-58527-0)
Supplement: Supplementary file 1 — Supplementary Materials. [file 41598_2020_58527_MOESM1_ESM.pdf]

Nano-vehicle for delivery and efficiency of anticancer auraptene against colon cancer cells

Nazila Jalilzadeh[a,c], Naser Samadi[b], , Roya Salehi[c,d*], Gholamreza Dehghan[a*], Mehrdad Iranshahi[e], Mohammad Reza Dadpour[f] , Hamed Hamishehkar[c]

[a] Faculty of Natural Sciences, University of Tabriz, Tabriz, Iran

[b] Department of Biochemistry and Clinical Laboratories, Faculty of Medicine, Tabriz University of Medical Sciences, Tabriz, Iran

[c] Drug Applied Research Center, Tabriz University of Medical Sciences, Tabriz, Iran

[d] Department of Medical Nanotechnology, Faculty of advanced medical sciences, Tabriz University of Medical Sciences, Tabriz, Iran

[e] Faculty of Pharmacy, Mashhad University of Medical Sciences, Mashhad, Iran

[f] Department of Horticulture, Faculty of Agriculture, University of Tabriz, Tabriz, Iran

Correspondence:

*Roya Salehi, Tel: +984133355921. Fax: +984133355789, Email: salehiro@tbzmed.ac.ir

*Gholamreza Dehghan, Email: Dehghan2001d@yahoo.com

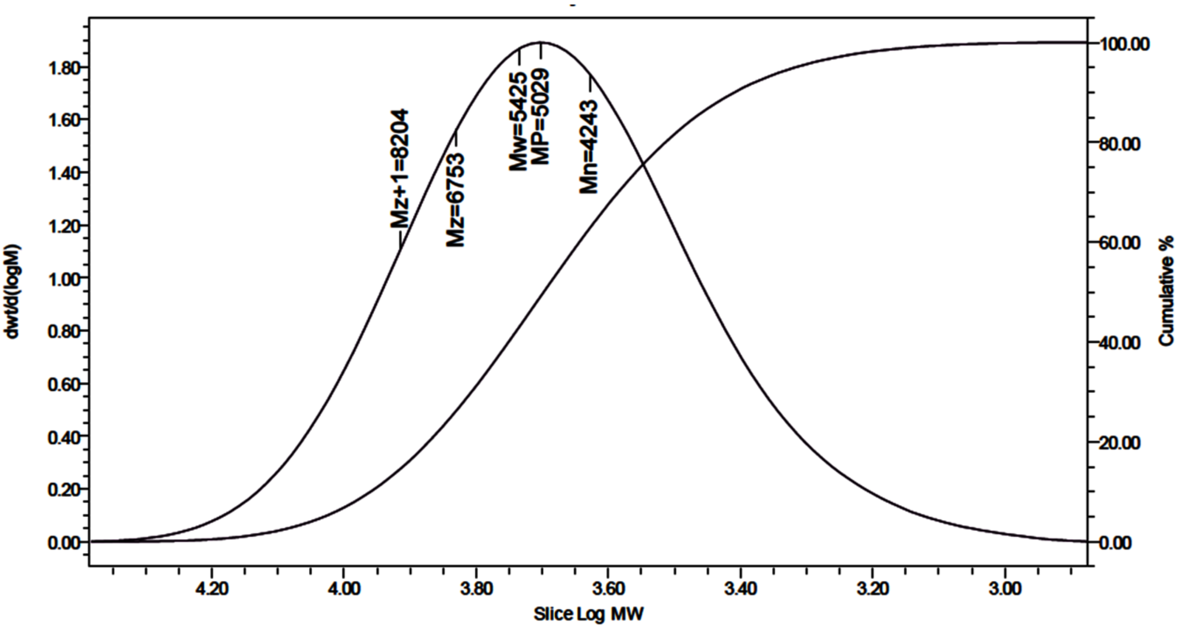

Figure S1. The number average (Mn) and weight average molecular (Mw) of the PCL-PEG-PCL (TB) copolymer determined by gel permission chromatography (GPC).

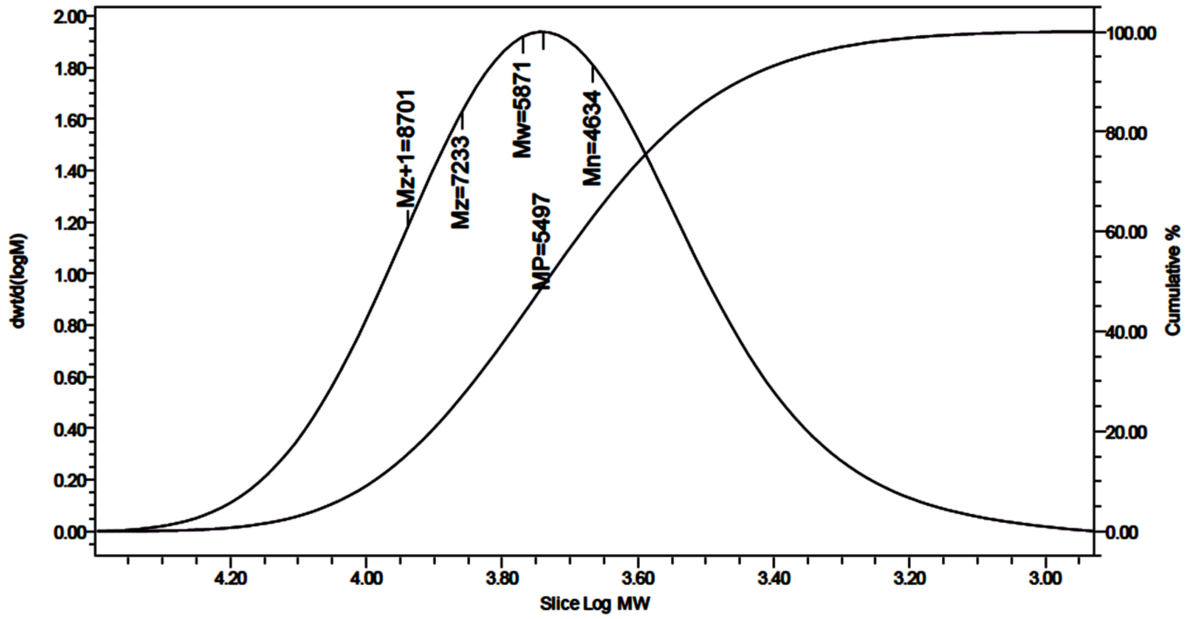

Figure S2. The number average (Mn) and weight average molecular (Mw) of the PLA-PCL-PEG-PCL-PLA (PB) copolymer determined by gel permission chromatography (GPC).

Results

PdI: 0.675

	Diam. (nm)	% Intensity	Width (nm)
Peak 1:	308.0	56.8	68.92
Peak 2:	78.99	43.2	26.53
Peak 3:	0.000	0.0	0.000

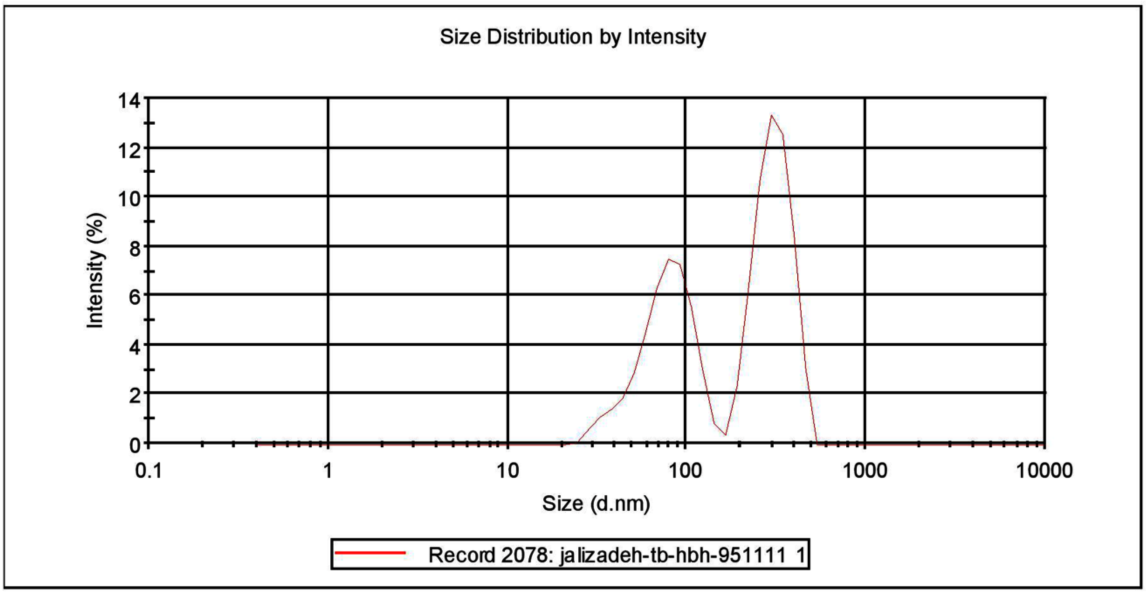

Figure S3. The average size of PCL-PEG-PCL triblock (TB) nanoparticles prepared by high pressure homogenization (HPH) method, determined by Dynamic Light Scattering (DLS) method.

.

Results

Zeta Potential (mV): -19.6

	Mean (mV)	Area (%)	Width (mV)
Peak 1:	-19.6	100.0	5.39
Peak 2:	0.00	0.0	0.00
Peak 3:	0.00	0.0	0.00

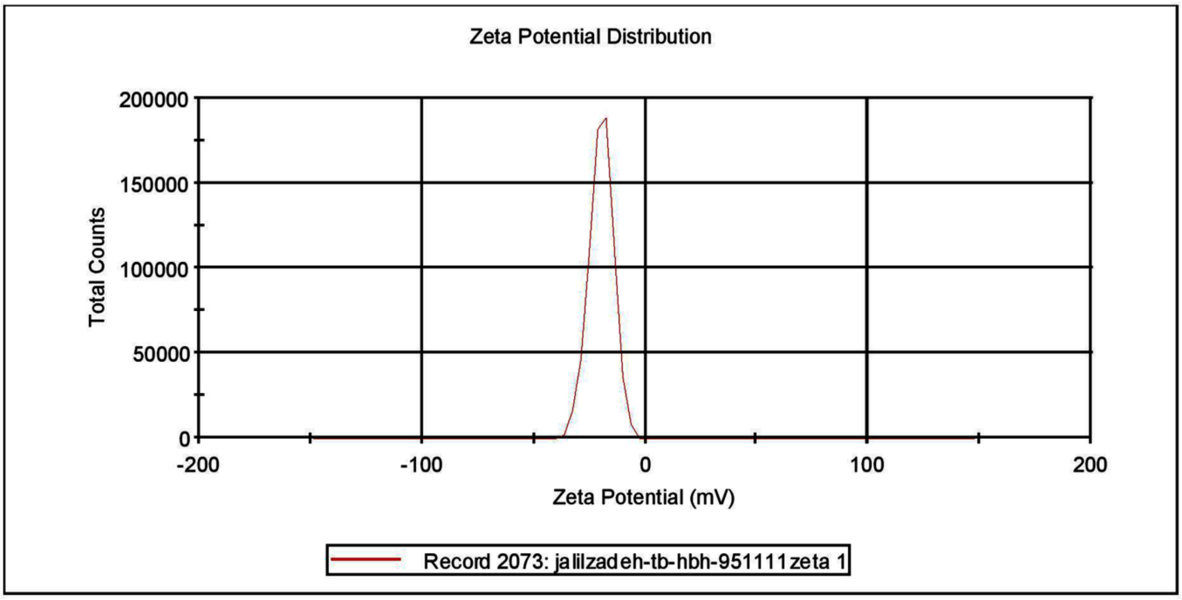

Figure S4. The Zeta potential of PCL-PEG-PCL triblock (TB) nanoparticles prepared by high pressure homogenization (HPH) method, determined by Zeta sizer.

Results

		Diam. (nm)	% Number	Width (nm)
	Peak 1:	314.5	100.0	76.11
PdI: 0.192	Peak 2:	0.000	0.0	0.000
	Peak 3:	0.000	0.0	0.000

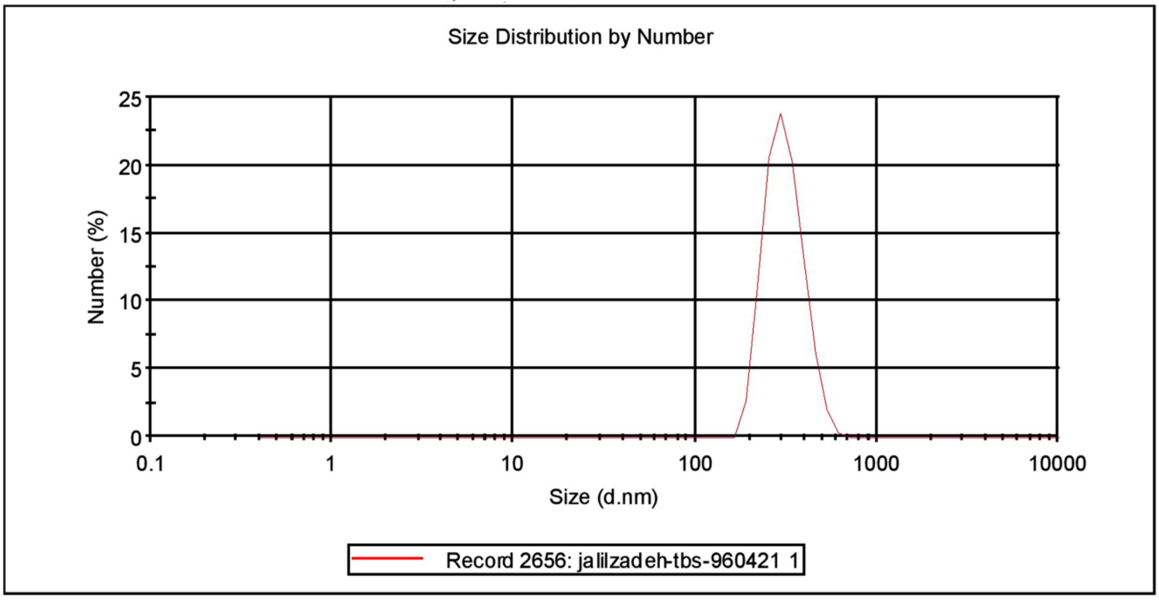

Figure S5. The average size of PCL-PEG-PCL triblock (TB) nanoparticles prepared by ultrasound (US) method, determined by Dynamic Light Scattering (DLS) method.

Results

Zeta Potential (mV): -9.26

	Mean (mV)	Area (%)	Width (mV)
**Peak 1:**	-9.26	100.0	3.91
**Peak 2:**	0.00	0.0	0.00
**Peak 3:**	0.00	0.0	0.00

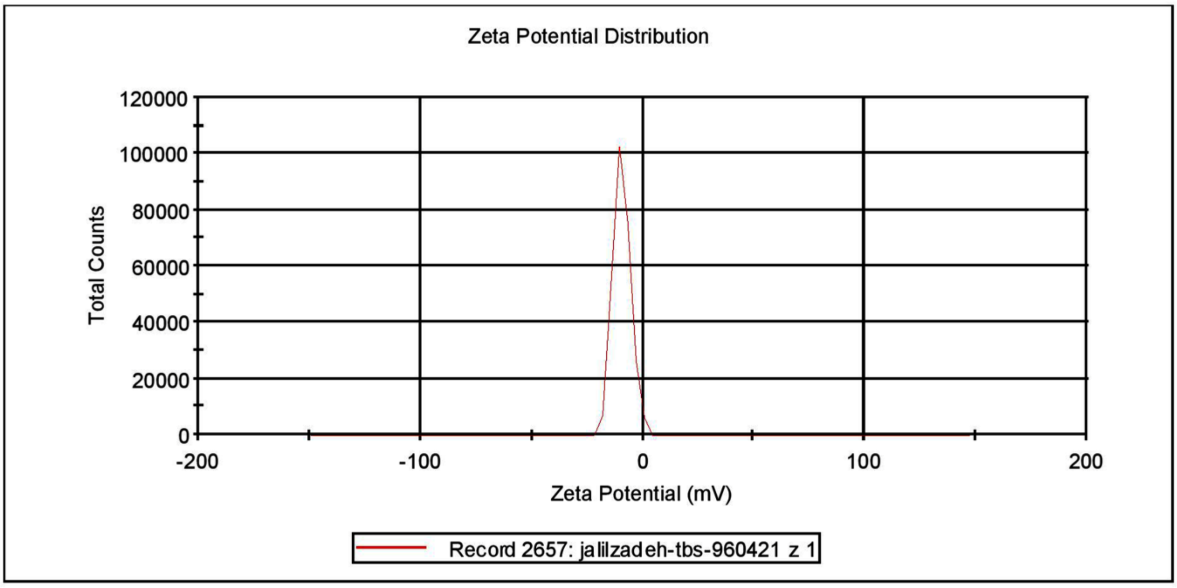

Figure S6. The Zeta potential of PCL-PEG-PCL triblock (TB) nanoparticles prepared ultrasound (US) method, determined by Zeta sizer.

Results

		Diam. (nm)	% Intensity	Width (nm)
	Peak 1:	170.3	100.0	17.11
PdI: 0.805	Peak 2:	0.000	0.0	0.000
	Peak 3:	0.000	0.0	0.000

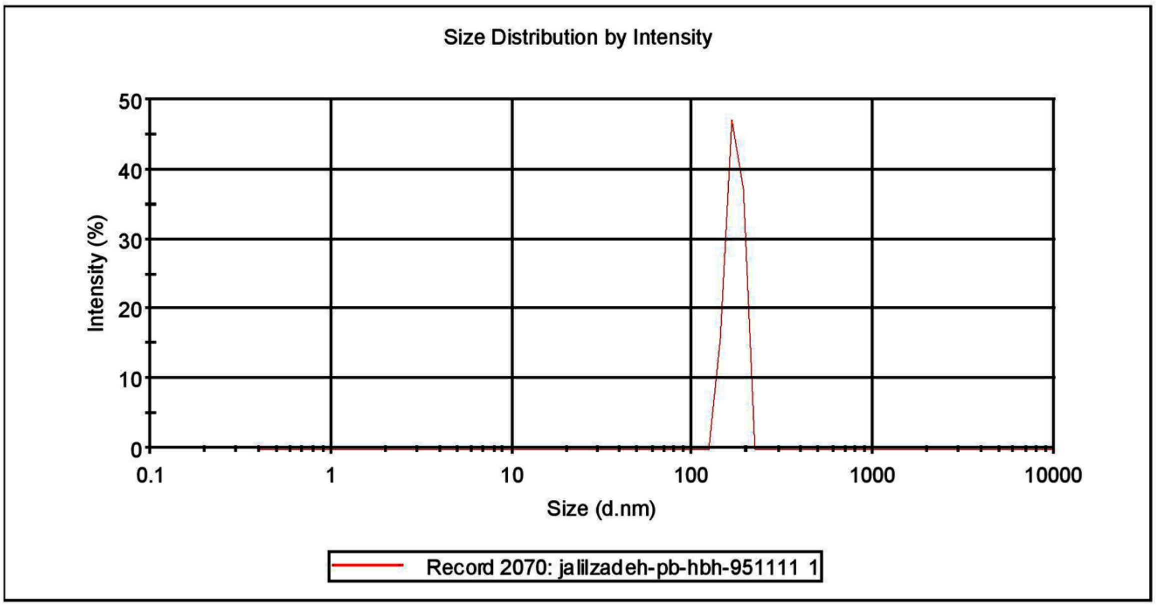

Figure S7. The average size of PLA- PCL-PEG-PCL-PLA (PB) nanoparticles prepared by high pressure homogenization (HPH) method, determined by Dynamic Light Scattering (DLS) method.

Results

Zeta Potential (mV): -26.4

	Mean (mV)	Area (%)	Width (mV)
**Peak 1:**	-21.1	84.9	5.61
**Peak 2:**	-37.9	15.1	3.84
**Peak 3:**	0.00	0.0	0.00

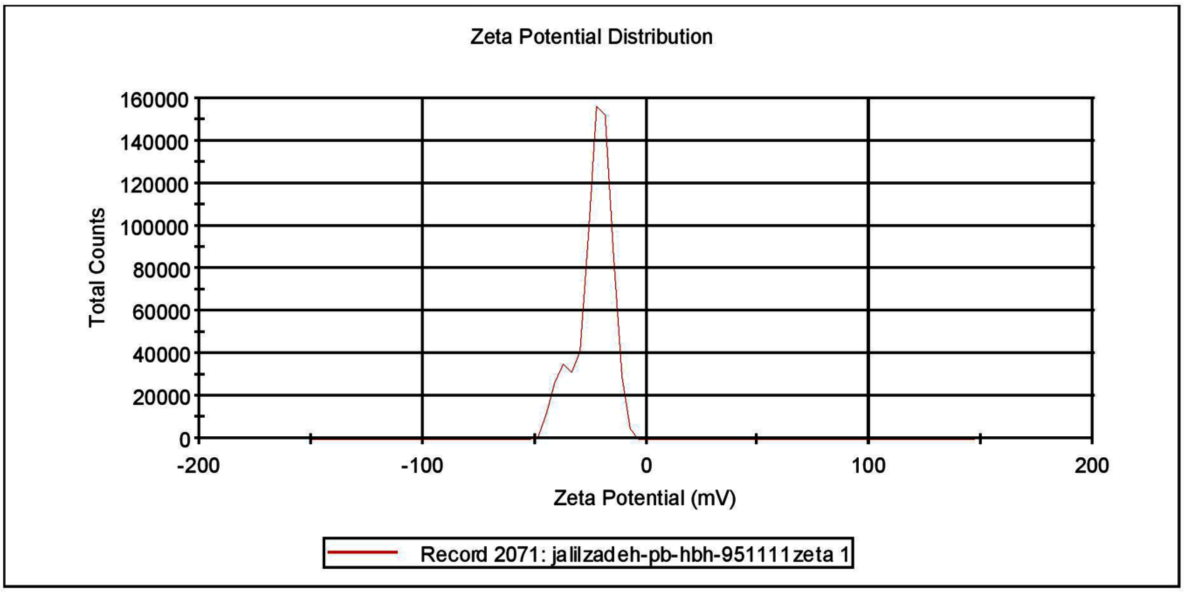

Figure S8. The Zeta potential of PLA- PCL-PEG-PCL-PLA (PB) nanoparticles prepared by high pressure homogenization (HPH) method, determined by Zeta sizer.

Results		Diam. (nm)	% Number	Width (nm)
	Peak 1:	109.8	100.0	31.79
PdI: 0.288	Peak 2:	0.000	0.0	0.000
	Peak 3:	0.000	0.0	0.000

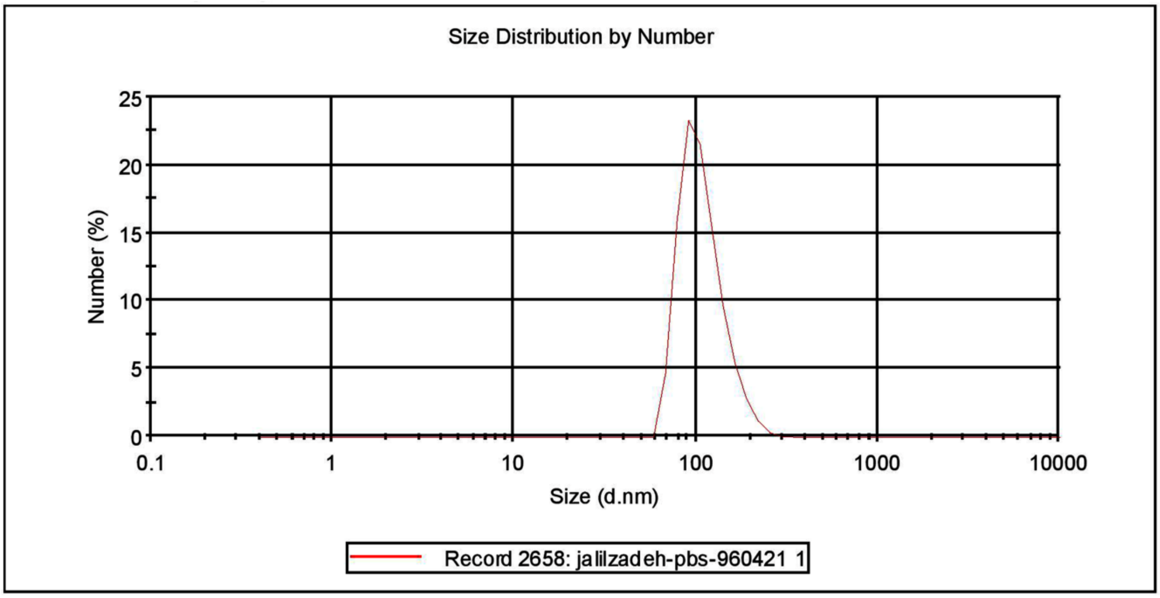

Figure S9. The average size of PLA- PCL-PEG-PCL-PLA (PB) nanoparticles prepared by ultrasound (US) method, determined by Dynamic Light Scattering (DLS) method.

Results

Zeta Potential (mV): -17.5

	Mean (mV)	Area (%)	Width (mV)
**Peak 1:**	-17.5	100.0	4.39
**Peak 2:**	0.00	0.0	0.00
**Peak 3:**	0.00	0.0	0.00

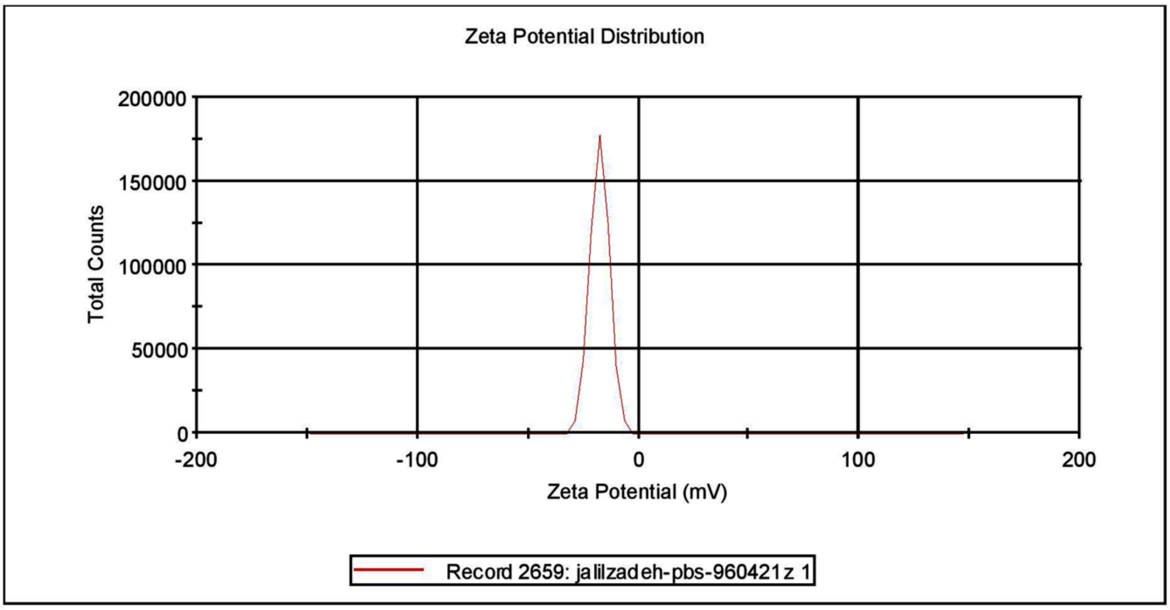

Figure S10. The Zeta potential of PLA- PCL-PEG-PCL-PLA (PB) nanoparticles prepared ultrasound (US) method, determined by Zeta sizer.

Results		Size (d.n...	% Intensity:	St Dev (d.n...
	Peak 1:	265.8	86.4	33.38
PdI: 0.758	Peak 2:	20.31	13.6	2.145
	Peak 3:	0.000	0.0	0.000

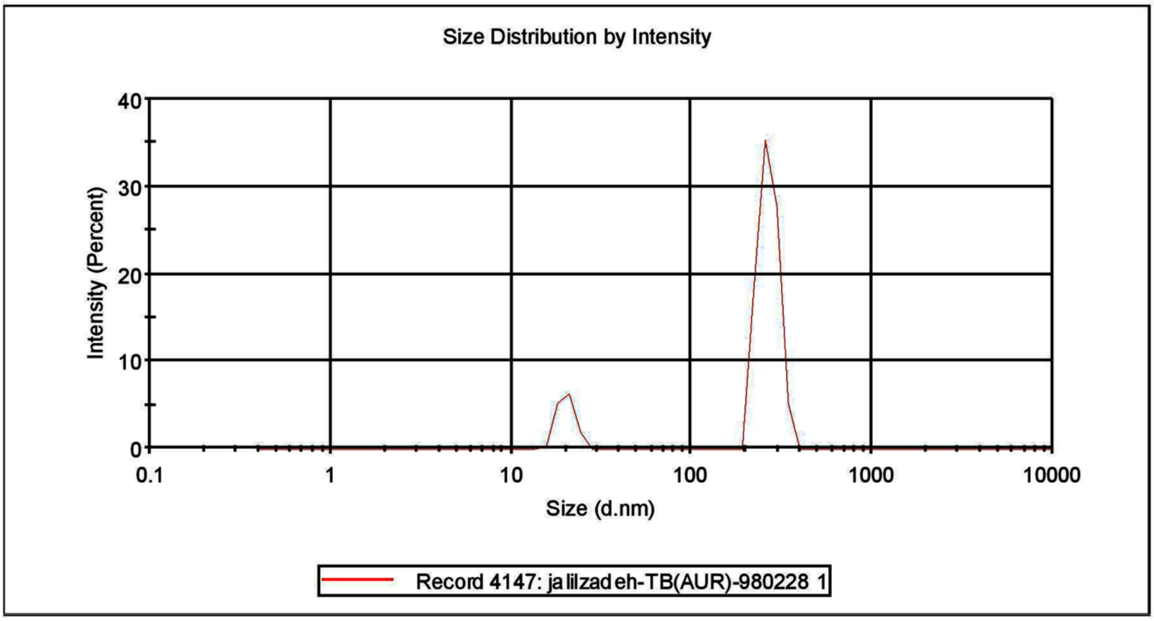

Figure S11. The average size of auraptene (AUR)-loaded PCL-PEG-PCL (TB) nanoparticles prepared by ultrasound (US) method, determined by Dynamic Light Scattering (DLS) method.

Results		Size (d.n...	% Number:	St Dev (d.n...
	Peak 1:	153.6	100.0	50.29
PdI: 0.533	Peak 2:	0.000	0.0	0.000
	Peak 3:	0.000	0.0	0.000

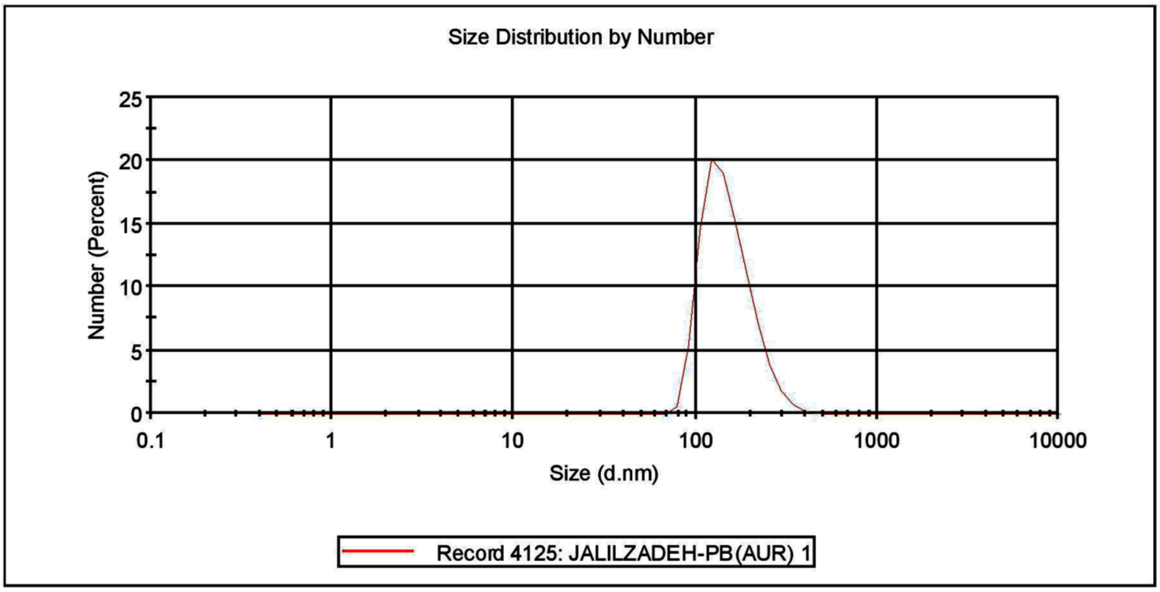

Figure S12. The average size of auraptene (AUR)-loaded PLA-PCL-PEG-PCL-PLA (PB) nanoparticles prepared by ultrasound (US) method, determined by Dynamic Light Scattering (DLS) method.

Results

		Mean (mV)	Area (%)	St Dev (mV)
	Peak 1:	-16.4	100.0	4.73
Zeta Potential (mV): -16.4	Peak 2:	0.00	0.0	0.00
	Peak 3:	0.00	0.0	0.00

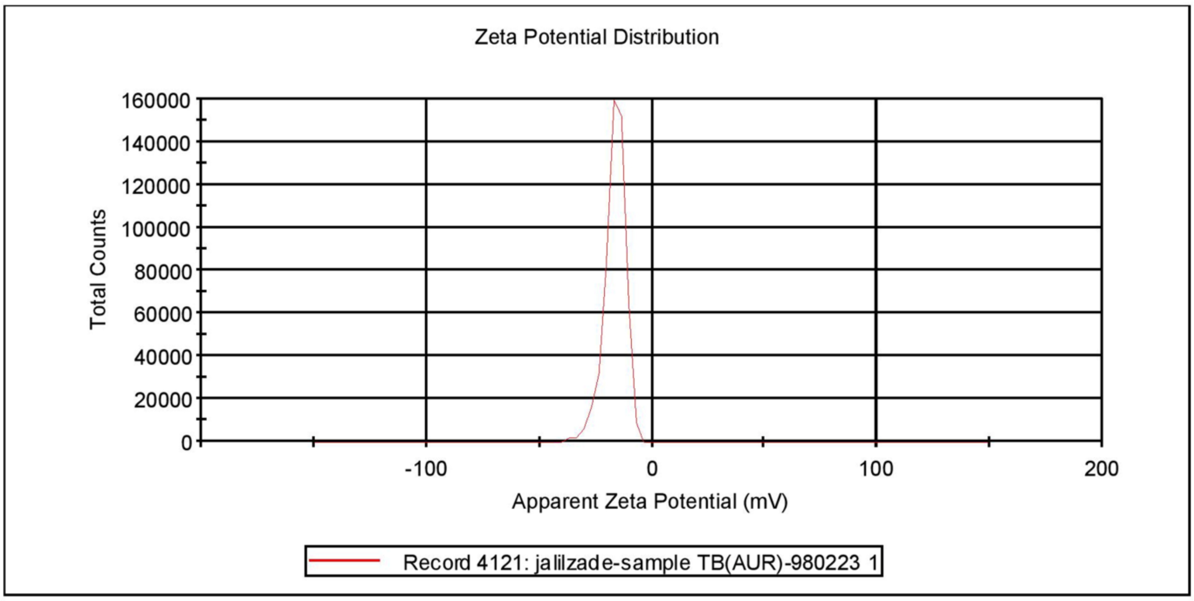

Figure S13. The Zeta potential of auraptene (AUR)-loaded PCL-PEG-PCL (TB) nanoparticles prepared by ultrasound (US) method, determined by Zeta sizer.

Results

Zeta Potential (mV): -15.0

	Mean (mV)	Area (%)	St Dev (mV)
**Peak 1:**	-11.8	80.0	4.89
**Peak 2:**	-29.8	19.5	4.01
**Peak 3:**	13.5	0.5	1.69e-7

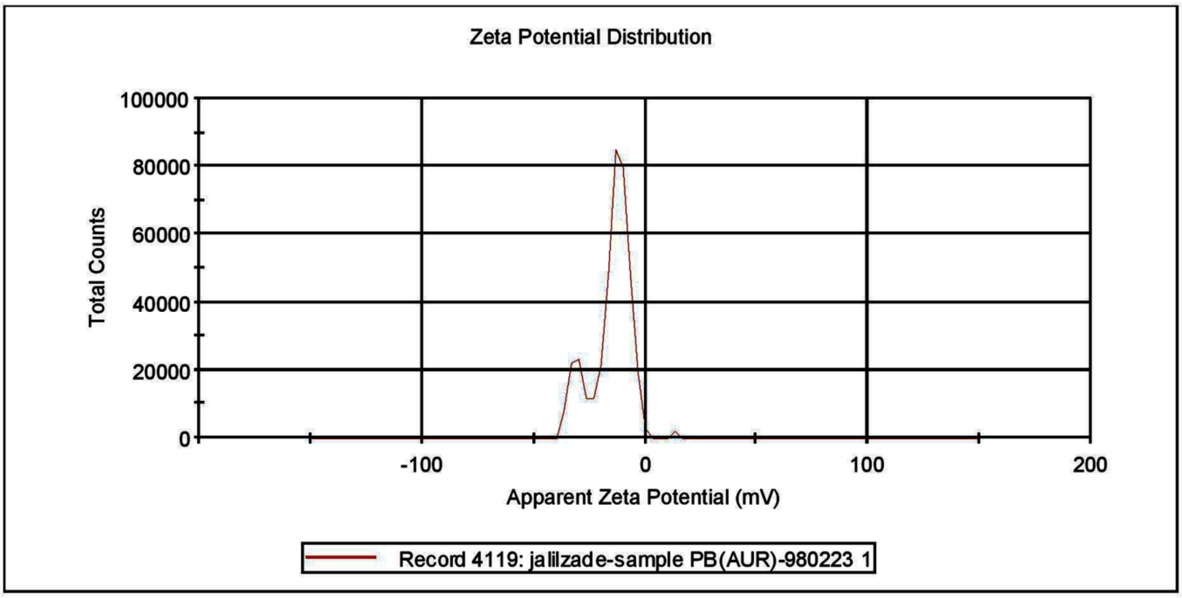

Figure S14. The Zeta potential of auraptene (AUR)-loaded PLA-PCL-PEG-PCL-PLA (PB) nanoparticles prepared by ultrasound (US) method,
